# Dioxygenation of tryptophan residues by superoxide and myeloperoxidase

**DOI:** 10.1016/j.jbc.2025.108402

**Published:** 2025-03-11

**Authors:** Nina Dickerhof, Louisa V. Ashby, Daniel Ford, Joshua J. Dilly, Robert F. Anderson, Richard J. Payne, Anthony J. Kettle

**Affiliations:** 1 Mātai Hāora - Centre for Redox Biology and Medicine, Department of Pathology and Biomedical Science, University of Otago Christchurch, Christchurch, New Zealand; 2 School of Chemistry, The University of Sydney, Sydney, New South Wales, Australia; 3 Australian Research Council Centre of Excellence for Innovations in Peptide and Protein Science, The University of Sydney, Sydney, New South Wales, Australia; 4 School of Chemical Sciences & Auckland Cancer Society Research Centre, The University of Auckland, Auckland, New Zealand

**Keywords:** neutrophil, tryptophan oxidation, phagosome, dioxygenase, hypochlorous acid, *N*-formylkynurenine, calprotectin, lactoferrin

## Abstract

When neutrophils ingest pathogens into phagosomes, they generate large amounts of the superoxide radical through the reduction of molecular oxygen. Superoxide is essential for effective antimicrobial defense, but the precise role it plays in bacterial killing is unknown. Within phagosomes, superoxide reacts with the heme enzyme myeloperoxidase (MPO) and is converted to hydrogen peroxide, then subsequently to the bactericidal oxidant hypochlorous acid. But other reactions of superoxide with MPO may also contribute to host defense. Here, we demonstrate that MPO uses superoxide to dioxygenate tryptophan residues within model peptides *via* two hypochlorous acid–independent pathways. Using mass spectrometry, we show that formation of *N*-formylkynurenine is the favored reaction. This reaction is consistent with a direct transfer of dioxygen from an intermediate of MPO, where superoxide is bound to the active site heme iron (compound III). In addition, hydroperoxides are formed when superoxide adds to tryptophan radicals, which are produced during the peroxidase cycle of MPO. Proteomic analysis revealed that tryptophan dioxygenation occurs on the abundant neutrophil protein calprotectin and lactoferrin during phagocytosis of *Staphylococcus aureus*, indicating that this is a physiologically relevant modification. Our study enhances the understanding of superoxide chemistry in the phagosome. It also suggests that tryptophan dioxygenation by MPO and superoxide may occur during infection and inflammation.

The capacity to generate large amounts of superoxide is an exceptional and defining characteristic of neutrophils. After these abundant innate immune cells ingest pathogens, superoxide is produced by the NADPH oxidase complex that is mobilized to the membranes of the ensuing phagosomes (1). Superoxide itself is not directly toxic to bacteria because of its low reactivity and limited cell permeability (1, 2, 3). It does, however, react in multiple ways with the heme enzyme myeloperoxidase (MPO), which is concurrently released into the phagosome (4). Whether these reactions occur during phagocytosis has yet to be established.

MPO is one of the most abundantly expressed proteins in neutrophils, accounting for 5% of the dry weight of the cell (5), highlighting its importance for immune function. It produces the potent bactericidal oxidant hypochlorous acid (HOCl) from hydrogen peroxide (6, 7). While production of HOCl to kill ingested pathogens is presumed to be its sole function in the phagosome, this assumption remains to be reconciled with the observation that HOCl reacts predominantly with neutrophil proteins rather than ingested bacteria (8, 9). Furthermore, depending on the microbe, HOCl contributes only partially or not at all to the microbial killing inside the phagosome (10, 11).

Superoxide produced in the phagosome first reacts rapidly with MPO to form compound III, also known as oxymyeloperoxidase. This redox intermediate can be envisaged as either superoxide coordinated to the ferric enzyme or dioxygen bound to the ferrous enzyme (Fig. 1) (4). Previous kinetic analysis suggested that compound III is then reduced further by superoxide in a slow reaction to give the ferric enzyme and hydrogen peroxide, which react rapidly with each other to form compound I (4). Compound I is the redox intermediate that oxidizes chloride to HOCl (Fig. 1). Superficially, the function of superoxide in this context is to act simply as a precursor for hydrogen peroxide. However, this nonessential role for superoxide raises the question of why the neutrophil is not equipped with an oxidase that generates hydrogen peroxide directly. Given the extraordinarily high concentrations achievable for both superoxide and MPO inside phagosomes, it is likely that much of their chemistry within this unique location remains to be fully appreciated (12).

**Figure 1:**
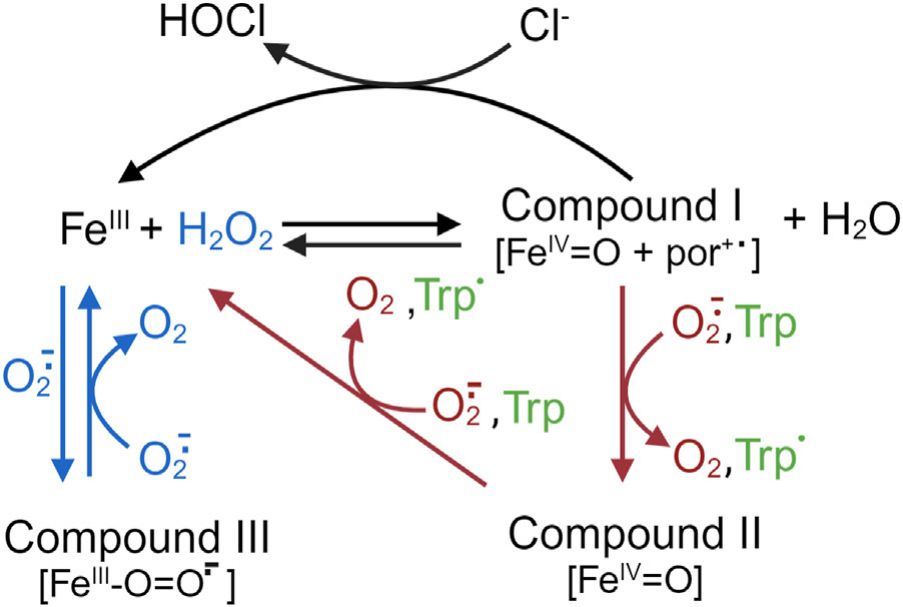
**Reactions of MPO with superoxide.** MPO acts as a superoxide dismutase (*blue*), when superoxide sequentially reacts with the enzyme's ferric state (Fe^III^) and the ferric superoxide intermediate compound III. MPO can also act as a superoxidase (*red*), using hydrogen peroxide to oxidize superoxide to oxygen *via* compounds I and II. This scheme was adapted from Ref. (4). Tryptophan (Trp) is oxidized to a tryptophan radical *via* the enzyme's peroxidase cycle. These reactions are in addition to its halogenation activity, whereby compound I oxidizes chloride to hypochlorous acid (HOCl). MPO, myeloperoxidase.

It has been proposed that superoxide maintains HOCl production in the phagosome by reducing MPO compound II back to the ferric (Fe^3+^) form (4). Compound II is formed upon reduction of compound I by numerous electron donors, and its accumulation would ultimately inhibit the halogenation cycle of MPO (Fig. 1). Yet employment of an HOCl-specific fluorescent probe has shown that phagosomal HOCl production is increased in the presence of superoxide dismutase (SOD) (13). This result indicated that superoxide might in fact dampen HOCl production, possibly by reacting with MPO to form compound III (Fig. 1). Paradoxically, oxidative killing was found to be significantly diminished when SOD was phagocytosed by neutrophils along with pathogens (14, 15, 16, 17). Collectively, these studies highlight a role for superoxide in the oxidative antimicrobial activity of neutrophils that is independent of HOCl production.

Previously, MPO was found to add dioxygen to melatonin *via* superoxide-dependent reactions, but the mechanisms of oxidation were not fully clarified (18). Superoxide may add to radicals generated by MPO compound I and compound II in the classic peroxidase cycle to form hydroperoxides (1, 19, 20, 21, 22). Moreover, when superoxide reduces compound I or II, it is thermodynamically feasible that the released molecular oxygen is in its reactive singlet state (23, 24). Singlet oxygen oxidizes tryptophan, tyrosine, histidine, and methionine residues, nucleobases, and unsaturated fatty acids (25, 26) and is lethal to bacteria. It has been proposed to be formed during phagocytosis, but the evidence is equivocal (7, 27, 28). In addition, compound III may be capable of directly oxygenating substrates held within the enzyme’s active site. This reaction mechanism is analogous to that of heme dioxygenase enzymes tryptophan 2,3-dioxygenase and indolamine-2,3-dioxygenase, which use molecular oxygen bound to the ferrous (Fe^2+^) enzymes to oxidize tryptophan to *N*-formylkynurenine (NFK) (29). Alternatively, compound III may oxidize substrates, as it does for ascorbate (30) and serotonin (31), to produce radicals that combine with a second molecule of superoxide to form hydroperoxides. A final possibility is that MPO compound III is reduced by superoxide to a compound I-like intermediate that hydroxylates substrates in a cytochrome P450–type mechanism (32).

The aim of the present study was to investigate reactions of superoxide with MPO and to determine whether they are involved in the oxygenation of substrates in an HOCl-independent manner, either by forming hydroperoxides, releasing singlet oxygen, or through direct oxygen transfer.

## Results

### Oxygen is added to the tryptophan residue of KWF in the presence of MPO and superoxide

**Figure 2:**
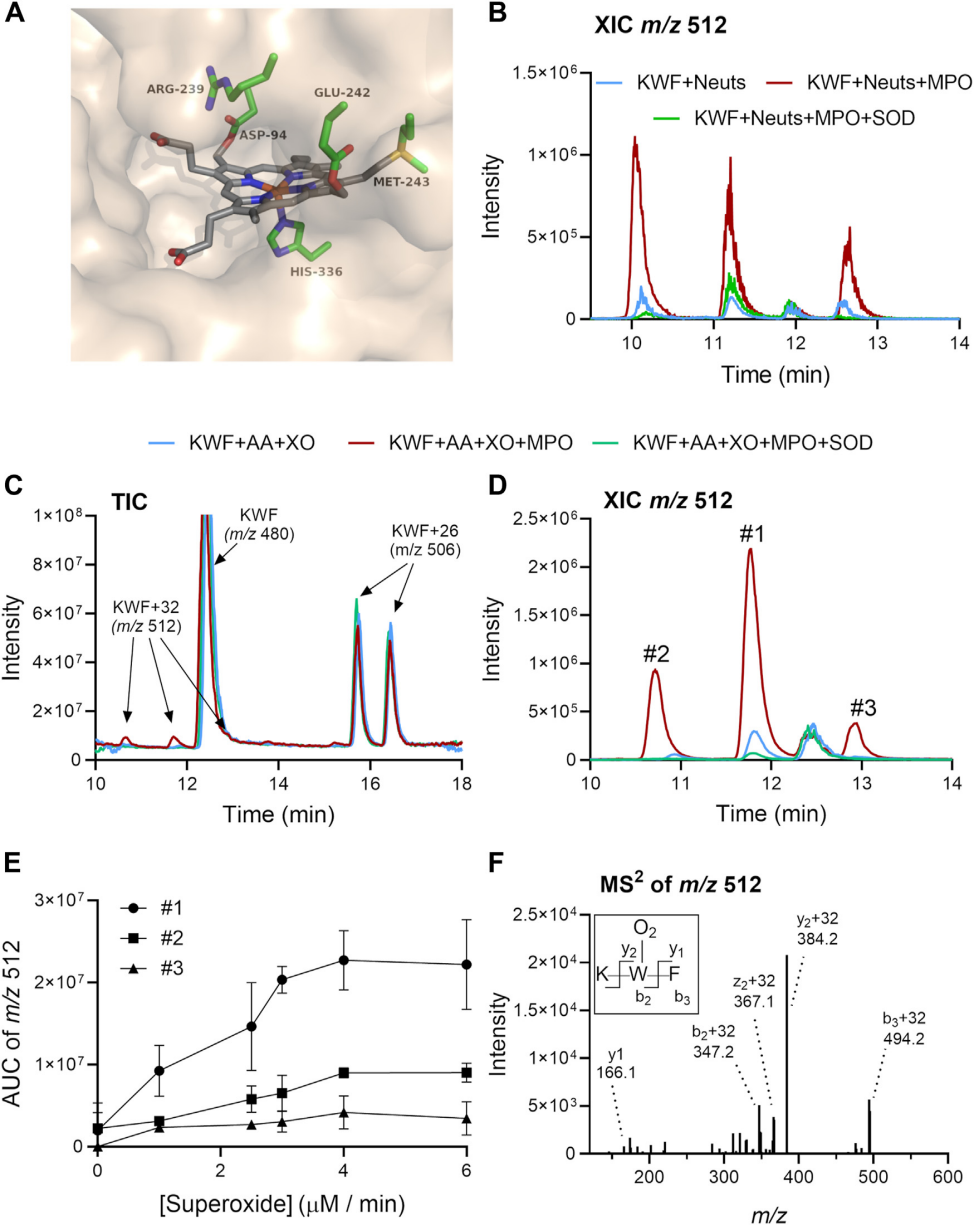
**Incorporation of oxygen into the tryptophan residue of peptide KWF by MPO and superoxide produced by stimulated neutrophils or xanthine oxidase (XO).***A*, the structure of the MPO active site pocket showing the heme prosthetic group (*gray*) with its negatively charged propionate groups on the *left* and covalent linkages to Asp94, Met243, Glu242, and His336. Atoms are colored by element: oxygen, *red*; nitrogen, *blue*; and sulfur, *yellow*. The figure was constructed in PyMOL using the coordinates deposited in the Protein Data Bank (accession code: 1CXP). *B*, peptide KWF (200 μM) and neutrophils (Neuts, 10^6^ cells/ml) were incubated with PMA (100 ng/ml) in the absence or the presence of MPO (100 nM) and superoxide dismutase (SOD) (20 μg/ml) in HBSS. After 30 min at 37 °C, neutrophils were removed by centrifugation. Products in the supernatants were separated on a Jupiter Proteo column and detected by LC–MS in positive ion mode. A representative extracted ion chromatogram (XIC) of three independent experiments is shown for *m/z* 512 representing the KWF peptide + 32 mass units. *C*, KWF (100 μM), acetaldehyde (AA, 10 mM), and XO (4 μM O_2_^.-^/min) were incubated in the absence or the presence of MPO (100 nM) and SOD (20 μg/ml) in 100 mM phosphate, pH 7.4 with 10 μM DTPA for 30 min at 22 to 24 °C. The reaction was stopped by adding SOD (20 μg/ml) and analyzed as in *B*. A representative total ion chromatogram (TIC) of at least five independent experiments is shown. *D*, a representative XIC is shown for *m/z* 512 representing the KWF peptide + 32 mass units. *E*, the superoxide flux was varied by varying the concentration of XO, and the area under the curve (AUC) for the three *m/z* 512 species #1, #2, and #3 was determined. *F*, a representative MS^2^ spectrum for the major *m/z* 512 species #1 eluting at 11.8 min. The MS^2^ spectrum for the two minor species #2 & #3 was similar. In all panels, the chromatograms and spectra represent at least three independent experiments. DTPA, diethylenetriaminepentaacetic acid; HBSS, Hank's buffered salt solution; KWF, lysine-tryptophan-phenylalanine; MPO, myeloperoxidase; MS, mass spectrometry; PMA, phorbol 12-myristate 13-acetate.

We focused our attention on tryptophan as a potential target for MPO-derived oxidants because tryptophan undergoes numerous reactions with oxidants to produce distinctive products (19, 33, 34, 35). We used tryptophan within small peptides, mainly lysine-tryptophan-phenylalanine (KWF), because tryptophan is a poor substrate for MPO (36) and is more likely to exist in the phagosomes within small peptides released during digestion of proteins rather than as the free amino acid. The slow reaction of free tryptophan with compound II of MPO may in part be the result of charge repulsion between its negatively charged α-carboxylate and that of the enzyme’s heme group in the active site cleft (Fig. 2*A*) (36, 37). We reasoned that the positively charged lysine residue in KWF would facilitate electrostatic interaction with the heme carboxylate, and that the phenylalanine would enhance binding to the largely hydrophobic distal heme pocket (38, 39).

We initially investigated whether neutrophils could dioxygenate tryptophan in the peptide KWF. Isolated neutrophils (1 × 10^6^ cells/ml) were stimulated with phorbol 12-myristate 13-acetate (PMA) in the presence of added MPO (100 nM) and KWF (200 μM). Under the conditions used here, neutrophils will on average generate an extracellular superoxide flux of about 8 μM/min (40). After 30 min, the supernatants were analyzed using mass spectrometry (MS) by selecting for the *m/z* value of the peptide (*m/z* 480) plus addition of two oxygen atoms (*m/z* 512). Three products with an *m/z* value of 512 were identified (Fig. 2*B*). Their formation was inhibited by SOD and diminished without the addition of MPO. These results suggest that neutrophils can use MPO and superoxide to add dioxygen to tryptophan residues within peptides.

**Figure 3:**
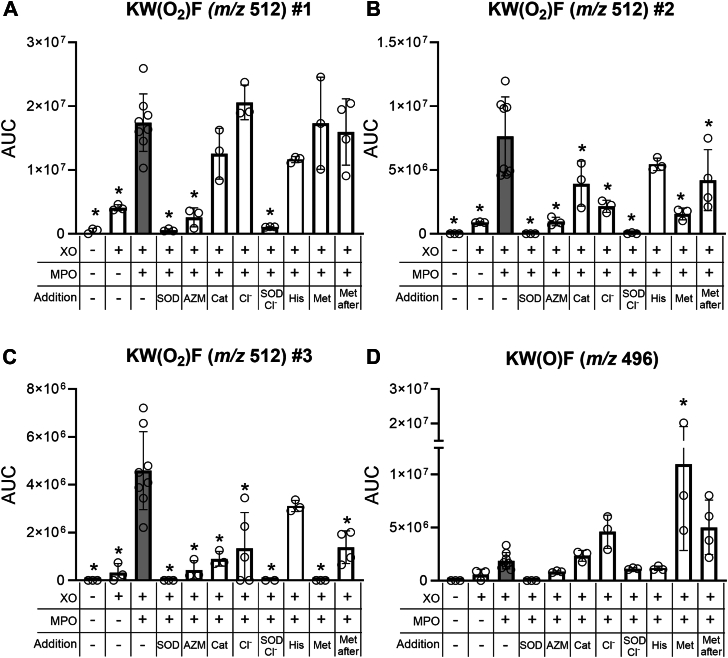
**Probing the mechanism for KWF dioxygenation using alternative substrates and oxidant scavengers.** Reaction conditions are as in Figure 2*C*, but the following inhibitors were added at the start of the 30 min incubation: superoxide dismutase (SOD, 20 μg/ml), MPO inhibitor AZM198 (AZM, 40 μM), catalase (Cat, 20 μg/ml), sodium chloride (Cl^-^, 100 mM), methionine (Met, 1 mM), and histidine (His, 1 mM). Met (1 mM) was also added at the end of the 30 min incubation (“after”) and incubated for another 30 min. The AUC was determined for *A*–*C*, the 3 *m/z* 512 species #1–3 and (*D*) an *m/z* 496 species representing the addition of one oxygen atom to the tryptophan. Data points represent independent experiments, and the bar represents the mean ± SD. A statistical difference compared to the “XO + MPO” system, that is, the *gray* bar, was determined by one-way ANOVA with Dunnett’s multiple comparisons test and is indicated by ∗ for *p* < 0.05. AUC, area under the curve; MPO, myeloperoxidase.

To investigate the mechanism of dioxygenation of KWF by MPO and superoxide in greater depth and to characterize the products, we switched to a xanthine oxidase (XO) system because it is more reliably manipulated than neutrophils. KWF was exposed to MPO in chloride-free buffer with a superoxide flux (4 μM O_2_^.-^/min) generated by XO and acetaldehyde. Reactions were stopped by the addition of SOD, and the products were monitored by LC–MS. In this reaction system, two major products with additional 26 mass units (*m/z* 506) eluting at 15.7 and 16.4 min were formed (Fig. 2*C*). These were also present in controls that did not contain XO or MPO and most likely correspond to imines resulting from the reaction of acetaldehyde with the N-terminal or ϵ-amine moiety of the lysine side chain of the peptide. As found for the neutrophil system, three smaller peaks eluted at 10.5, 11.7, and 12.8 min, all of which had an *m/z* value of 512 (Fig. 2, *C* and *D*). These findings are consistent with KWF gaining 32 mass units (*m/z* 512) and indicate that dioxygen was added to the peptide. The dioxygenated products formed in the XO system were labeled #1 to 3 based on their relative intensities. Formation of the +32 species was not observed when MPO was omitted from the reaction mixture or when SOD was added to remove superoxide (Figs. 2, *C* and *D* and 3, *A*–*C*). Adding catalase or the XO inhibitor allopurinol (400 μM) in conjunction with SOD to stop reactions was no more effective than adding SOD alone (data not shown). The yield of the dioxygenated products was dependent on the rate of superoxide generation (Fig. 2*E*), suggesting that superoxide is required in a rate-determining step in product formation. The MS^2^ spectrum of all *m/z* 512 species confirmed that dioxygen was added to the tryptophan as the y_1_ ion containing only the phenylalanine residue was unmodified, whereas the y_2_ ion composed of both tryptophan and phenylalanine residues carried the +32 modification (Fig. 2*F*). Thus, we conclude that MPO can use superoxide, either generated by neutrophils or XO, to add molecular oxygen to tryptophan residues within peptides.

### MPO dioxygenates tryptophan of KWF to NFK and hydroperoxides

To probe which reaction mechanisms were responsible for the three observed +32 products of KWF, we added a panel of inhibitors, alternative substrates, and oxidant scavengers to the reaction mixture containing MPO, acetaldehyde, and XO (Fig. 3). AZM198, a specific 2-thioxanthine type inhibitor of MPO that covalently binds to the heme prosthetic group of the enzyme, inhibited the formation of all three +32 species (Fig. 3, *A*–*C*). In contrast, catalase, which removes hydrogen peroxide, thus preventing formation of compound I, decreased the yield of just the two minor +32 products #2 and #3 (Fig. 3, *A*–*C*). Similarly, only the major product #1 was unaffected by the addition of chloride (Fig. 3, *A*–*C*), which acts as a substrate for compound I, reducing MPO back to its ferric state and generating HOCl in the process (Fig. 1). None of the +32 products were formed when SOD was added with chloride (Fig. 3, *A*–*C*). This system predominantly generates HOCl, because SOD will convert all the superoxide to hydrogen peroxide, which in turn is used by MPO to oxidize chloride. Likewise, dioxygenation did not occur when reagent HOCl was added to KWF instead of the enzyme system as discussed later. Collectively, these results indicate that formation of the major tryptophan dioxygenation product was independent of both hydrogen peroxide and HOCl. The minor products were also independent of HOCl but most likely required radical formation *via* compound I.

Next, we added histidine to the reaction mixture to probe the involvement of diffusible singlet oxygen released from MPO during its redox cycle. Due to a threefold higher rate constant for the reaction of histidine with singlet oxygen compared with tryptophan (41), histidine should outcompete KWF at the 10-fold molar excess used here. Histidine had no significant effect on the formation of any of the +32 products (Fig. 3, *A*–*C*), ruling out diffusible singlet oxygen as the mediator of tryptophan dioxygenation.

Since formation of the two minor +32 products #2 and #3 was dependent on hydrogen peroxide as well as superoxide, and was inhibited by chloride, it is likely that these products are hydroperoxides. They can be formed from the addition of superoxide to a tryptophan radical generated when KWF is oxidized by compound I. To support this proposal, we determined whether KWF could inhibit MPO-dependent HOCl production by reducing compound I and converting the enzyme to compound II (Fig. 1). We found that KWF inhibited MPO by 50% at a concentration of 2.3 μM (Fig. S1). Inhibition was substantially decreased by addition of tyrosine to the reaction system to promote turnover of compound II as previously shown for tryptophan (Fig. S1) (36). Thus, we conclude that tryptophan-containing peptides can reduce compound I to compound II. In the process, they would be oxidized to a radical that can react with superoxide to form a hydroperoxide.

To provide further evidence that products #2 and #3 are hydroperoxides, we tested whether their signal was decreased in the presence of reductant methionine, which is expected to reduce hydroperoxides to their respective alcohols (42, 43). When methionine was present in the reaction mixture with KWF, MPO, and XO, signals of the minor +32 products #2 and #3 were significantly lower compared with the control (Fig. 3, *B* and *C*). The major product #1 was unaffected by methionine (Fig. 3*A*). To rule out the possibility that methionine’s effect was mediated through scavenging a reactant, rather than reduction of the hydroperoxide products, we also added methionine after the reaction was allowed to proceed for 30 min. Methionine was still able to decrease the level of the two minor +32 products when added at the end of the reaction (Fig. 3, *B* and *C*). Furthermore, the signal of an alcohol species (+16 mass units, *m/z* 496) eluting at 10 min was increased in the presence of methionine (Fig. 3*D*), consistent with it acting as a reductant on the hydroperoxide products #2 and #3.

**Figure 4:**
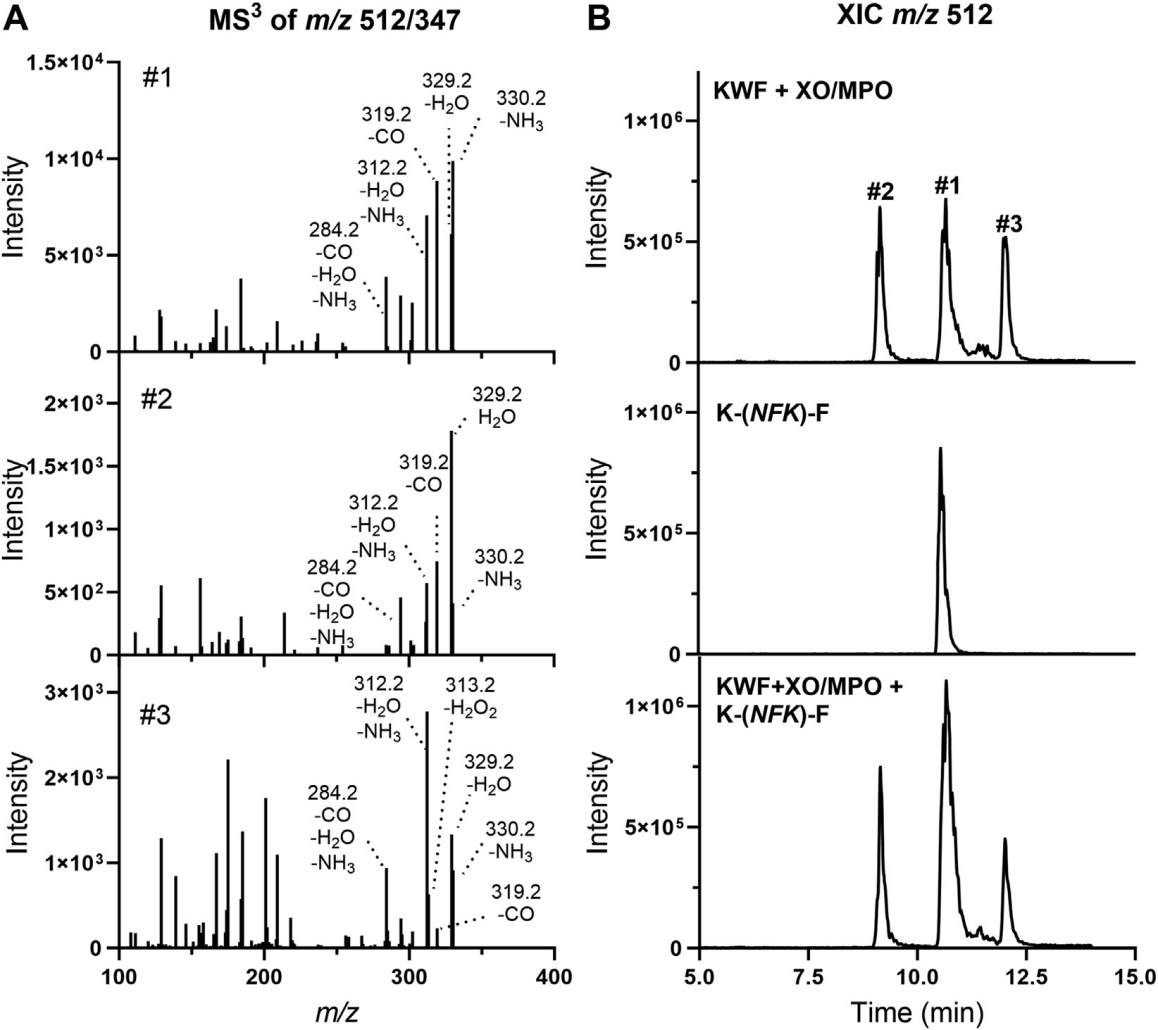
**Determining the structure of the KWF dioxygenation products using LC–MS^3^ and an analytical K-*NFK*-F standard.***A*, the b_2_ (*m/z* 347.2) ions in the MS^2^ spectra of all three *m/z* 512 species representing KWF +32 (shown for the major product #1 in Figure 2*F*) were subjected to further fragmentation. Representative MS^3^ spectra are shown. The numbers #1, #2, and #3 represent the products shown in Figure 2*D*. *B*, extracted ion chromatograms (XICs) for *m/z* 512 following injection of the full enzyme system comprised of XO, MPO, acetaldehyde, and KWF (100 μM) described in Figure 2*D* (*top*), an *N*-formylkynurenine (NFK) containing peptide K-NFK-F peptide standard (10 μM) (*middle*) and a mixture of the two (*bottom*). KWF, lysine-tryptophan-phenylalanine; MPO, myeloperoxidase; MS, mass spectrometry; XO, xanthine oxidase.

To support our conclusions from the inhibitor studies, we obtained structural information for the +32 products using MS^3^ experiments. The b_2_ + 32 (*m/z* 347.2) and the y_2_ + 32 (*m/z* 384.2) ions formed during the MS^2^ run shown in Figure 2*F* were subjected to further fragmentation. The resulting MS^3^ spectra observed for all three +32 products were similar to each other and showed predominantly neutral losses of 18 (H_2_O), 17 (NH_3_), and 28 (CO) (Figs. 4*A* and S2). However, there were subtle differences in the relative intensities of the fragment ions consistent with small differences in the molecular arrangement and the presence of functional groups in the parent ion. For example, loss of NH_3_ and CO was observed to a greater degree for the b_2_ and y_2_ ions of major +32 product (#1), with the loss of CO suggesting the presence of a formyl group. Loss of CO in combination with NH_3_ was previously reported to be the favored fragmentation pathway for NFK (34). A neutral loss of 34 was detected in one of the two minor +32 products (#3 in Figs. 4*A* and S2). This represents the loss of H_2_O_2_ and is indicative of a hydroperoxide (34, 44).

The yield of the major dioxygenation product in the XO–MPO enzyme system was too low to allow for purification and direct characterization by NMR spectroscopy. Therefore, to establish whether it contained an NFK residue, we chemically synthesized the K-*NFK*-F peptide to use as a standard in our LC–MS analyses. First, Fmoc-Trp(Boc)-OH was reacted to form NFK through oxidative cleavage by treatment with ozone and dimethyl sulfide (Fig. S3) (45). The protected NFK amino acid was then incorporated into a K*-NFK*-F peptide using solid-phase peptide synthesis. When analyzed by LC–MS, the K-*NFK*-F peptide behaved in an identical manner to the major +32 product of KWF, that is, it coeluted with product #1 when spiked into the KWF–XO–MPO product mixture (Fig. 4*B*) and showed the same fragmentation pattern (Fig. S4). This result indicates that analogous to heme dioxygenases that use oxygen to oxidize tryptophan to NFK (29), MPO can generate the same tryptophan dioxygenation product within peptides by employing superoxide.

### Effects of KWF on formation and decay of compound III

**Figure 5:**
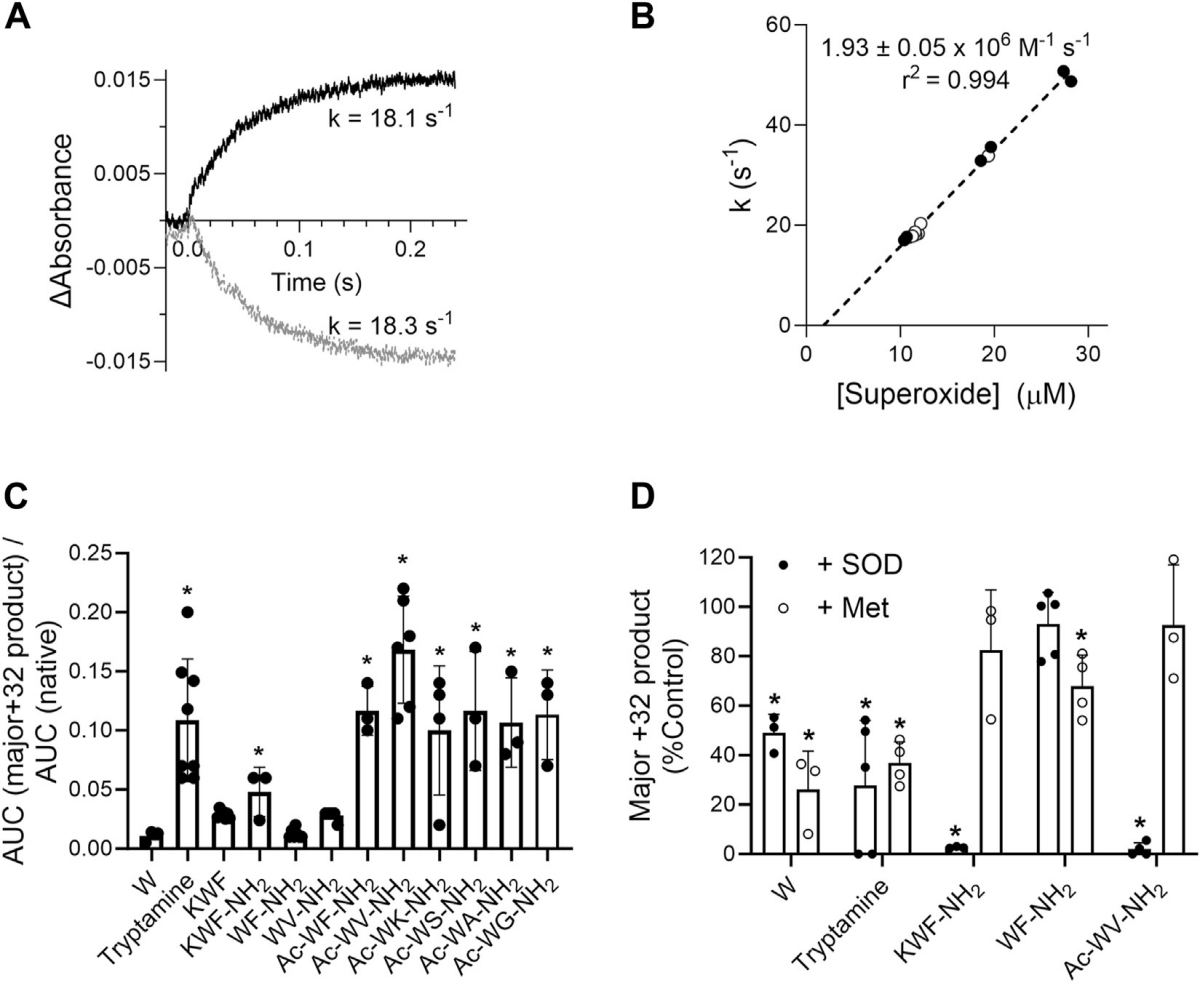
**Investigating the dependence of tryptophan dioxygenation on MPO compound III and substrate structure.** MPO (0.72 μM) was added to oxygenated 25 mM phosphate buffer (pH 7.4) containing 100 mM formate and 20 μM DTPA at 25 °C. The solutions were then pulsed with 20.5 to 52.9 Gy to give between 10 and 28 μM superoxide, and the reactions were monitored at 430, 452, or 625 nm. *A*, a representative trace of the changes in absorbance at 452 nm (*black*) and 430 nm (*gray*) when superoxide reacts with MPO. *B*, the first-order rates of the reactions were determined and plotted against the concentration of superoxide. Reactions were carried out in the absence (*open circles*, n = 7) or the presence of 200 μM KWF (*closed circles*, n = 6). The slope of the line was determined by liner regression analysis (n = 13, *p* < 0.0001). *C*, tryptophan (W), tryptamine, and tryptophan-containing peptides were incubated with MPO and xanthine oxidase/acetaldehyde as described for KWF in Figure 2*C*. The AUC for the major +32 product of each peptide/amino acid was expressed as a ratio relative to the native peptide/amino acid. Data points represent independent experiments, and the bar represents the mean ± SD. A statistical significance (*p* < 0.05) was determined by one-way ANOVA with Dunnett's multiple comparisons test and is indicated by ∗ for comparisons to KWF. *D*, SOD (20 μg/ml, *closed circles*) or methionine (Met, 1 mM, *open circles*) were included in the reaction, and the AUC of the +32 product was expressed relative to that formed in the absence of inhibitor (%Control). A statistical significance (*p* < 0.05) compared with the 100% control was determined separately for each peptide/amino acid by one-way ANOVA with Dunnett's multiple comparisons test and is indicated by ∗. AUC, area under the curve; DTPA, diethylenetriaminepentaacetic acid; KWF, lysine-tryptophan-phenylalanine; MPO, myeloperoxidase; SOD, superoxide dismutase.

Because the formation of NFK (product #1) in KWF by MPO was not inhibited by catalase (Fig. 3), it most likely involved MPO compound III. This is the predominant redox form of the enzyme in superoxide-generating systems that contain catalase to prevent formation of compound I (4, 46). To investigate possible mechanisms involving compound III, we interrogated the stability of compound III in the presence of KWF. First, we generated superoxide using pulse radiolysis and monitored changes in the absorbance of MPO. The redox intermediates of MPO have distinct absorption spectra that can be used to track its transformations. The ferric enzyme has a Soret absorbance peak at 430 nm, whereas compound II and compound III have broader Soret peaks at 452 nm, with another distinct but smaller peak at 625 nm that is more pronounced for compound III (4). Formate was present at a high concentration in oxygenated buffer to ensure all radical species were converted to superoxide. Formate is also a substrate for compound I and reduces it back to the ferric enzyme in a two-electron reaction (k = 2.8 × 10^4^ M^−1^ s^−1^) (4). When MPO was subjected instantaneously to excess superoxide by pulse radiolysis, there was a first-order loss in absorbance at 430 nm with a kinetically equivalent increase in absorbance at 452 nm (Fig. 5*A*). The ratio of absorbance changes at 452 and 625 nm (ΔA_625_/ΔA_452_ = 0.69) were typical of compound III (46). When SOD was added to the reaction system, there were no discernible absorbance changes as SOD would have converted all the superoxide to hydrogen peroxide, which would react with the ferric enzyme to form compound I. Under our reaction conditions, formate would rapidly recycle compound I and prevent any formation of compound II (4). Thus, the absorbance changes we monitored in the absence of SOD can be attributed only to formation of compound III. The first-order rate constants calculated from the change in absorbance were proportional to the initial concentration of superoxide (Fig. 5*B*). The second-order rate constant for reaction of superoxide with the ferric enzyme is given by the slope of this line and was 1.93 ± 0.05 × 10^6^ M^−1^ s^−1^. This value is close to what was measured previously for human and equine MPO (4, 47). When KWF (200 μM) was included in the reaction system, there was no change in the rate of conversion of ferric MPO to compound III (Fig. 5*B*). Thus, if KWF reacts with compound III, the rate constant must be at least an order of magnitude less than that for formation of compound III (Fig. 5*B*). We also gave MPO solutions two successive pulses of superoxide separated by a minute. In the first pulse, compound III would be produced and fully present when the solution was pulsed again. Under these conditions, we observed no changes in the absorbance of compound III. Thus, we must conclude that if superoxide reacts with compound III, the reaction is too slow to be observed by pulse radiolysis.

In addition to the pulse radiolysis experiments, compound III was produced using XO and acetaldehyde in the presence of chloride and catalase. Methionine was included to scavenge any HOCl. Compound III was allowed to accumulate until it reached a steady state level and then KWF (1 mM) was added. KWF had no effect on the stability of compound III (results not shown). This result agrees with the pulse radiolysis data.

### Dioxygenation of tryptophan by MPO depends on the peptide sequence

If tryptophan dioxygenation involves binding of the peptides to MPO, then variations in the peptide sequence should impact the product yields. In contrast, if oxidation occurs *via* a freely diffusible MPO-generated oxidant, then the extent of product formation is less likely to be dependent on the peptide sequence. Therefore, we modified the structure of the tryptophan-containing peptides and determined the extent of dioxygenation in the presence of MPO and a superoxide flux. As expected, the dioxygenation yield for free tryptophan was threefold lower than for KWF, but this did not reach statistical significance (Fig. 5*C*). The +32 products of tryptophan were reducible by methionine pointing to hydroperoxides. Interestingly, SOD only partially inhibited product formation (shown for the major product in Fig. 5*D*). Oxygen incorporation in the absence of superoxide might be the result of tryptophan being oxidized to a radical by MPO compound I, with subsequent addition of dissolved oxygen.

The dioxygenation yield increased significantly to 10-fold that of tryptophan when using tryptamine (Fig. 5*C*), a tryptophan analog lacking the carboxylic group. Only one peak consistent with +32 adduct formation on tryptamine was detected. However, this peak eluted at the solvent front, and, given the poor column retention, we suspect that it contained multiple products. Formation of the composite tryptamine +32 peak was superoxide dependent and partially inhibited by methionine (Fig. 5*D*), possibly indicating the presence of a mixture of both the radical-mediated hydroperoxides and the enzyme-catalyzed non-reducible product.

Analogously, oxygen incorporation into KWF could be increased twofold when the peptide’s C-terminal carboxylic group was replaced with an amide group, thus minimizing repulsion with the heme group’s negative charges (KWF *versus* KWF-NH_2_, Fig. 5*C*). The observation that the presence of a negatively charged group diminishes the reactivity toward the substrate is consistent with a previous study investigating the oxidation of aliphatic thiols by MPO (38). Interestingly, we achieved the greatest dioxygenation yields, that is, 3.5–6-fold when compared with KWF, when in addition to amide replacement, the N-terminal amine of the peptide was acetylated (WF-NH_2_ *versus* Ac-WF-NH_2_ and WV-NH_2_ *versus* Ac-WV-NH_2_, Fig. 5*C*). This may be the result of minimizing charge repulsion with MPO’s active site arginine 239 (Fig. 2*A*).

The major +32 product of all the peptides tested here was largely resistant to reduction by methionine (Fig. 5*D*). Except for the small, charged dipeptides WF-NH_2_ and WV-NH_2,_ incorporation of oxygen into peptides was superoxide dependent as revealed by complete inhibition by SOD (shown for KWF-NH_2_, WF-NH_2_, and Ac-WV-NH_2_ in Fig. 5*D*). As for tryptophan, oxygen incorporation into the small dipeptides might also take place *via* an alternative mechanism involving dissolved oxygen.

### Chloride-dependent products of tryptophan oxidation by MPO

When chloride was included in the reaction mixtures containing XO, acetaldehyde, and MPO, further KWF oxidation products were observed with additional 24 and 16 mass units compared with KWF (Fig. S5, *A*–*C*). These are likely the result of HOCl-dependent formation of a dehydro species of the acetylated peptide (+26, −2) and of tryptophan hydroxylation (+16). In support of this conclusion, +16 adduct formation also occurred upon treatment with reagent HOCl (Fig. S6*A*), but the +24 product was only observed when KWF was exposed to HOCl in the presence of acetaldehyde (Fig. S6*B*). Importantly, dioxygenation (+32) was not facilitated by reagent HOCl.

The HOCl-dependent hydroxylated species are distinct from the +16 species formed from the reduction of hydroperoxides as they eluted at different retention times (11.1 and 11.7 min *versus* 10 min, respectively). A hydroxyindole, which exists as a tautomer with the oxindole (Fig. S5*C*), is a known product for the reaction of HOCl with indoles and is proposed to be formed *via* hydrolysis of an initial 3-chloroindole (48, 49, 50). Indeed, we detected two peptides with *m/z* values of 514 and 516 consistent with chlorination (+34 and + 36) that coeluted at 13.2 min (Fig. S5, *A* and *D*). The two peptides showed the characteristic 3:1 isotope ratio representing ^35^Cl and ^37^Cl, which is indicative of a chlorinated species (Fig. S5*E*). The MS^2^ spectrum of *m/z* 514 and 516 confirmed that the addition of 34 and 36 mass units occurred on the tryptophan as both the y_2_ and b_2_ ions carried the modification (data not shown). Hydroxylated and chlorinated products were significantly lower when SOD was added with chloride (Fig. S5, *C* and *D*), most likely because of the requirement for superoxide to reduce compound II back to the ferric enzyme to maintain the MPO reaction cycle (Fig. 1). Additional chlorinated products with the characteristic 3:1 ratio that were not detected in the MPO system were formed when KWF was treated with HOCl reagent (Fig. S6*A*). These species warrant further investigation.

### Dioxygenation occurs on tryptophan residues of neutrophil proteins during phagocytosis

Next, we explored whether MPO-mediated tryptophan dioxygenation occurs within neutrophil phagosomes. We performed proteomic analyses on tryptically digested whole lysates and supernatants from neutrophils after they phagocytosed opsonized *Staphylococcus aureus* for 2 h. Using high-resolution, high mass accuracy Orbitrap MS and database searches using Proteome Discoverer, we identified on average 1993 ± 148 (mean ± SD, n = 3) proteins (false discovery rate <1%) based on 7763 ± 405 peptide groups (false discovery rate <5%) in the neutrophil lysates. A complete list of protein and peptide matches for each independent experiment can be found in the (Exp 1 Lysate.xlsx, Exp 2 Lysate.xlsx, Exp 3 Lysate.xlsx).

We filtered the search results for any peptides containing a dioxygenated tryptophan residue, and those meeting the following criteria were included as dioxygenation candidates: (i) The peptide confidence was at least medium (false discovery rate of <5%); (ii) The protein from which the peptide was derived had high confidence (false discovery rate of <1%); and (iii) The corresponding nonoxygenated peptide was also detected. Several peptides meeting these criteria were detected in lysates from neutrophils that have phagocytosed *S. aureus* but not unstimulated neutrophils (Table 1). Of these, two dioxygenated peptides, LTW(O_2_)ASHEK and GADVW(O_2_)FK, derived from calprotectin's subunits S100A9 and S100A8, respectively, were consistently detected in whole lysates from stimulated neutrophils from three different donors (Table 1). The nonoxidized counterparts of these dioxygenated peptides were detected in both stimulated and unstimulated neutrophils, but the dioxygenated forms were only found upon the addition of *S. aureus*, suggesting that neutrophil stimulation was required for their formation (Table 1).

**Table 1:** Dioxygenated peptides detected in neutrophil lysates and extracellular fractions after phagocytosis of *S. aureus*

Fraction	Peptide	Protein	*z*	*m/z*	Unstim	+*S. aureus*		
						Ctrl	+AZM	+DPI
Intracellular	LTW(O_2_)ASHEK	S100A9	2	502.245	✗✗✗	✗✓✓	✗✗✓	✗✗✓
			3	335.167	✗✗✗	✓✓✓	✓✗✓	✗✗✓
	GADW(O_2_)FK	S100A8	1	854.411	✗✗✗	✗✗✗	✗✓✗	✗✗✗
			2	427.706	✗✗✗	✓✓✓	✓✓✓	✗✗✓
	SLMFMQW(O_2_)GQLLDHDLDFTPEPAAR	MPO	3	950.777	✗✗✗	✓✗✗	✗✗✗	✗✗✗
	DSPSVW(O_2_)AAVPGK	Profilin-1	2	623.308	✗✗✓	✗✓✓	✗✓✗	✗✓✗
	LASDLLEW(O_2_)IR	Alpha-actinin-1	2	624.338	✗✗✗	✓✓✗	✓✗✗	✓✗✗
	VNDDIIVNW(O_2_)VNETLR	Plastin-2	2	916.463	✗✗✓	✓✓✗	✓✓✗	✓✗✗
			3	611.311	✗✗✗	✓✓✗	✓✗✗	✓✗✗
	YVDW(O_2_)R	Kallikrein-12	2	442.219	✗✗✗	✗✗✗	✓✗✗	✗✗✗
	SPSVEGSLW(O_2_)AVGTESQGR	Paxillin	3	626.967	✗✗✗	✓✗✗	✗✗✗	✗✗✗
	VALYDW(O_2_)IR	Myeloblastin	2	583.814	✗✗✗	✗✓✗	✗✗✗	✗✗✗
	IHECQW(O_2_)VVEDAPNPDVLLSHK	Protein Niban 1	3	821.065	✗✗✗	✗✗✗	✗✗✗	✓✗✗
Extracellular	EDAIW(O_2_)NLLR	Lactoferrin	2	581.300	✗✗✗	✓✓✓	✓✗✗	✓✗✗
	SVNGKEDAIW(O_2_)NLLR		3	549.622	✗✗✗	✗✓✓	✗✓✓	✗✓✗
	DVTVLQNTDGNNNEAW(O_2_)AK		2	1010.957	✗✗✗	✗✓✗	✗✗✗	✗✗✗
	SDTSLTW(O_2_)NSVK		2	635.300	✗✗✗	✓✓✗	✗✗✗	✗✗✗
	W(O_2_)LPAEYEDGFSLPYGWTPGVK	MPO	3	815.387	✓✗✗	✓✗✓	✗✗✗	✗✗✗
	W(O2)LPAEYEDGFSLPYGWTPGVKR		3	867.471	✗✗✗	✗✓✓	✗✗✗	✗✗✗
	VALYDW(O_2_)IR	Myeloblastin	2	583.811	✗✗✗	✗✗✗	✓✗✗	✗✗✗
	GADW(O2)FK	S100A8	2	427.706	✗✗✓	✗✗✗	✓✗✓	✗✗✗
	LTW(O_2_)ASHEK	S100A9	3	335.166	✗✗✓	✗✗✗	✗✓✗	✗✓✗
	AFKAW(O_2_)AVAR	Albumin	3	351.194	✗✗✗	✗✗✓	✗✗✗	✗✗✗

Neutrophils (10^7^/ml) were incubated with or without MPO and NADPH oxidase inhibitors, AZM198 (10 μM) and DPI (10 μM), respectively; and opsonized *S. aureus* or buffer (unstim) at 37 °C with end-over-end rotation for 2 h. Peptides containing a dioxygenated tryptophan residue (+31.9988 Da) identified in the whole lysate (intracellular) and extracellular fractions are shown alongside their *m/z*, charge *z*, and the protein they derived from. Peptides identified with medium/high confidence are indicated by a *tick*, and those absent or matched with low confidence by a *cross*. Each *symbol* denotes an independent experiment using blood from a different donor. The *shading* indicates that a peptide was either never detected (*white*), always detected (*dark gray*), or inconsistently detected (*light gray*).

**Figure 6:**
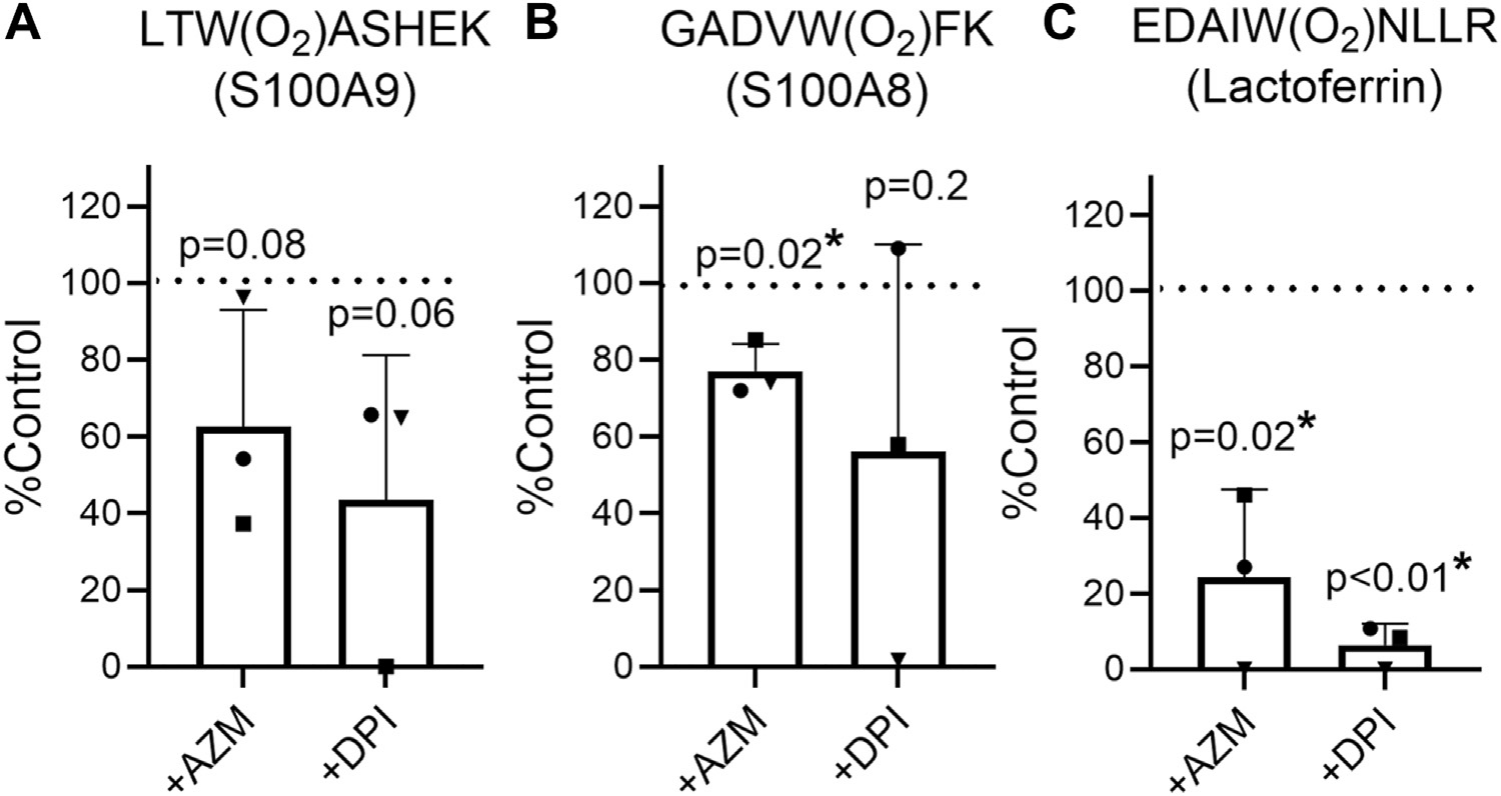
**Tryptophan dioxygenation occurs during neutrophil phagocytosis.** Neutrophils (10^7^/ml) in the absence (control) or the presence of 10 μM of the MPO inhibitor AZM198 or the NADPH oxidase inhibitor DPI were mixed with opsonized *Staphylococcus aureus* and incubated at 37 °C with end-over-end rotation for 2 h. The extracellular fractions and clarified neutrophil lysates were subjected to tryptic digestion followed by Orbitrap LC–MS/MS analysis. Database searches identified the following peptides containing dioxygenated tryptophan residues: (*A*) LTW(O_2_)ASHEK from S100A9, (*B*) GADVW(O_2_)FK from S100A8 in neutrophil lysates, and (*C*) EDAIW(O2)NLLR from lactoferrin in the extracellular sample. The area under the curve (AUC) of these peptides was obtained from extracted ion chromatograms, divided by the AUC of the respective unmodified peptides, and then expressed as a percentage of control. *Symbols* represent independent experiments using a different blood donor, with the bar denoting the mean + SD. The *p* value compared with the control (100%, *dotted line*) was determined by a one-tailed, one-sample *t* test and is indicated by ∗ for *p* < 0.05. DPI, diphenyleneiodonium; MPO, myeloperoxidase.

To demonstrate that the NADPH oxidase and MPO were required for the dioxygenation of LTWASHEK and GADVWFK, we inhibited the activity of these enzymes using diphenyleneiodonium (DPI) and AZM198, respectively, and determined the area under the curve for the dioxygenated peptide relative to the nonoxygenated peptide from extracted ion chromatograms. Upon inhibition of MPO and the NADPH oxidase, the signal for LTW(O_2_)ASHEK decreased on average by 38% and 79%, respectively (Fig. 6*A*). However, these results did not achieve statistical significance, likely because of the variable response of neutrophils from different blood donors. GADVW(O_2_)FK formation was significantly inhibited by 23% with AZM198 (Fig. 6*B*).

We also performed proteomic analyses on the extracellular fractions collected in our experiments because neutrophils release proteins into the medium during phagocytosis (51). In this analysis, 645 ± 136 (mean ± SD, n = 3) proteins were identified based on 2306 ± 794 peptide groups (Exp 1 Extracellular.xlsx, Exp 2 Extracellular.xlsx, Exp 3 Extracellular.xlsx). The dioxygenated peptide EDAIW(O_2_)NLLR was detected in the extracellular fraction in all three experiments (Table 1). It is derived from lactoferrin, which is known to be released into phagosomes and extracellularly by phagocytosing neutrophils (52, 53, 54). The signal for EDAIW(O_2_)NLLR significantly decreased by 76% and 94% upon addition of AZM198 and DPI, respectively (Fig. 6*C*), indicating that superoxide and MPO activity were required for its formation. Similarly, the dioxygenated W(O_2_)LPAEYEDGFSLPYGWTPGVK from MPO or its miscleaved variant W(O2)LPAEYEDGFSLPYGWTPGVKR was always present in the extracellular fraction of phagocytosing control but not AZM- or DPI-treated neutrophils (Table 1). Due to the variability in signal detection between the two peptide variants, reliable quantification was not possible. There was no consistent evidence for dioxygenation of tryptophan residues in bacterial peptides.

Collectively, our results from the proteomics experiments are consistent with an MPO and superoxide-dependent dioxygenation process occurring during neutrophil phagocytosis.

## Discussion

Neutrophils generate large amounts of superoxide when they ingest a pathogen, but the role of this oxygen-free radical in the phagosome has remained enigmatic. In the present investigation, we found multiple interactions of superoxide with the abundant neutrophil enzyme MPO that are relevant to bacterial killing in the phagosome and may be implicated in inflammatory signaling. Importantly, we show that MPO uses superoxide as a substrate to incorporate molecular oxygen into peptide-bound tryptophan residues. In addition, we have confirmed that superoxide reacts rapidly with ferric MPO to generate compound III and is required to recycle compound II formed upon reduction of compound I by small tryptophan-containing peptides.

Dioxygenation of tryptophan residues was not because of HOCl because it occurred in chloride-free solutions, and reagent HOCl did not generate dioxygenated products when reacted with KWF. Reagent HOCl did form hydroxylated species in addition to novel chlorinated species. However, the hydroxylated products were not the same as those formed *via* the superoxide-dependent mechanism because they had differing chromatographic properties. It is also unlikely that Fenton chemistry contributed to dioxygenation of tryptophan residues in our systems. Although superoxide slowly disproportionates to form dioxygen and hydrogen peroxide, which in the presence of divalent transition metals can promote Fenton-like oxidation reactions, the reaction of MPO with superoxide is 10-fold faster than the spontaneous disproportionation (rate constants of 1.9 × 10^6^ M^−1^ s^−1^ *versus* 2 × 10^5^ M^−1^ s^−1^, respectively) (4, 55). Hence, hydroxyl radical formation will not be a favored reaction in our systems. Indeed, Winterbourn (56) showed that MPO inhibits both hydrogen peroxide and hydroxyl radical formation from superoxide.

**Figure 7:**
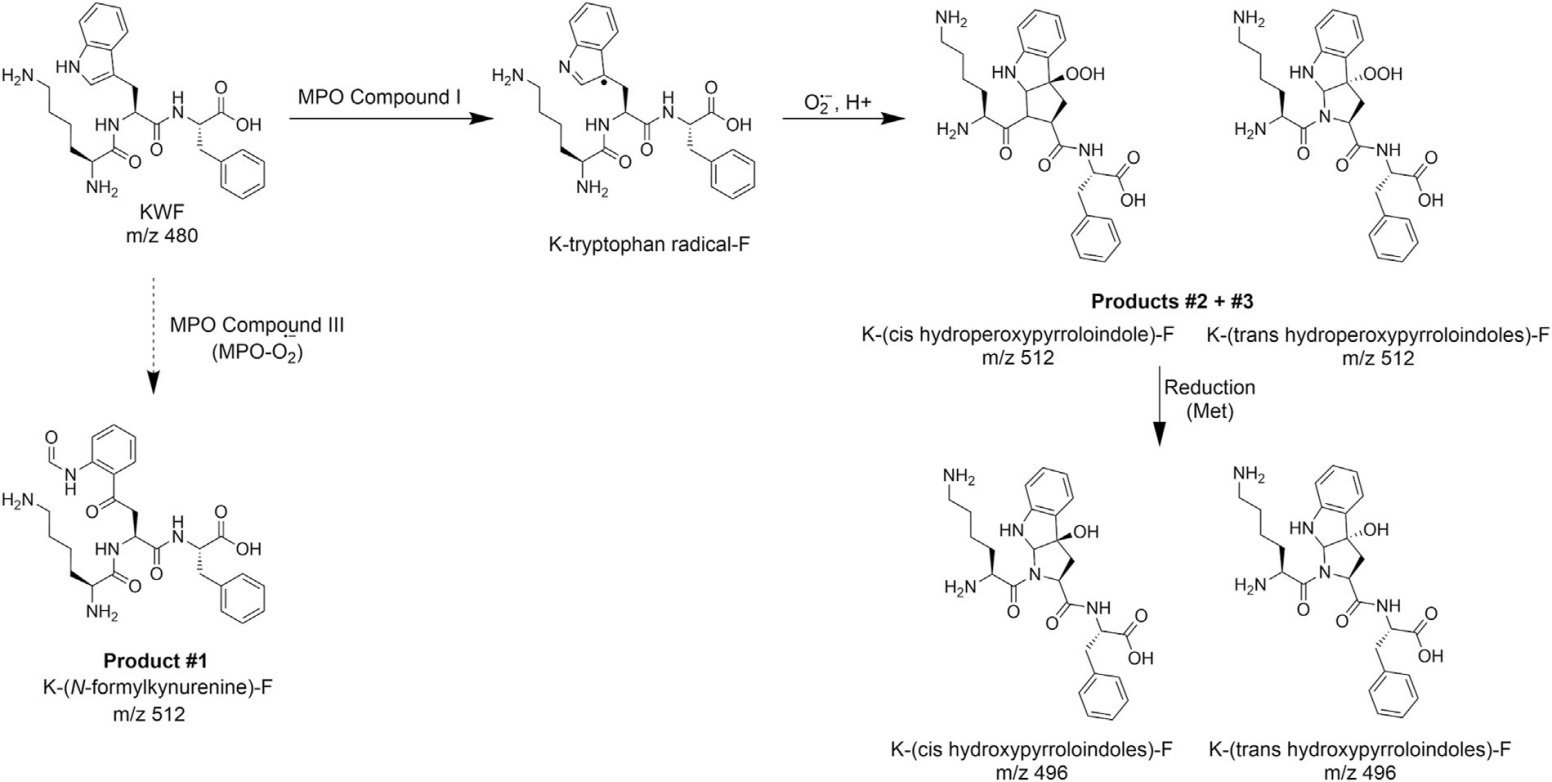
**Proposed reaction scheme for the dioxygenation of tryptophan-containing peptides by MPO and superoxide.** Superoxide reacts with ferric MPO to form the oxygenated ferric compound III (Fig. 1), which can transfer heme-bound dioxygen to the tryptophan of tryptophan-containing peptides such as KWF to form *N*-formylkynurenine. This mechanism is consistent with the formation of product #1 of the superoxide- and MPO-dependent oxygenation of KWF observed in Figure 2*D*. Alternatively, compound III can be reduced to compound I by reacting with another superoxide molecule (Fig. 1), which can oxidize the tryptophan of KWF to a radical intermediate. Subsequent addition of superoxide results in the formation of reducible hydroperoxides. These are consistent with products #2 & #3 observed in Figure 2*D*. KWF, lysine-tryptophan-phenylalanine; MPO, myeloperoxidase.

Our investigations provide insight into the reaction mechanism and the nature of the products formed when MPO dioxygenates tryptophan bound within peptides. Hydroperoxides are formed when superoxide adds to tryptophan radicals produced in the classic peroxidase cycle involving compound I and compound II (Fig. 7). We have also discovered a previously unrecognized tryptophan dioxygenase activity of MPO, most likely involving compound III of the enzyme, which leads to the formation of NFK (Fig. 7).

The mechanism by which tryptophan residues were oxidized in the presence of MPO depended on the peptide structure, with free tryptophan and small charged peptides being oxidized only to a small degree. These products were reducible suggesting that they were hydroperoxides formed *via* the radical-mediated mechanism. Formation of the nonreducible dioxygenation product was associated with greater yields but was only observed for predominantly uncharged peptides. This finding is consistent with the predicted affinity of the peptide to the MPO heme pocket, providing support for a mechanism that involves peptide binding.

Our data suggest that if the tryptophan-containing peptide KWF reacts with compound III, the reaction must be slow and the rate-determining step in dioxygenation. At this stage, it is not possible to conclude whether dioxygenation occurs *via* direct addition of dioxygen to tryptophan residues from compound III (MP^3+^:O_2_^*•*-^) in an analogous manner to heme dioxygenases (29), whereby ferrous MPO (MP^2+^) would be recycled back to compound III by molecular oxygen, as shown in reactions 1 and 2.

(1) MP^3+^:O_2_^*•*-^ + KWF → MP^2+^ + K-*NFK*-F

(2) MP^2+^ + O_2_ → MP^3+^:O_2_^*•*-^

Our attempts to demonstrate that ferrous MPO is produced in reaction 1 were frustrated by the need to have oxygen present to form compound III but absent to observe formation of the reduced enzyme.

Alternatively, dioxygenation may occur *via* a concerted mechanism in which compound III oxidizes tryptophan residues to radicals that add superoxide and then decay to the resultant NFK-containing peptide, as shown in reactions 3 and 4.

(3) MP^3+^:O_2_^*•*-^ + KWF → Compound I + K-W^*•*^-F

(4) K-W^*•*^-F + O_2_^*•*-^ → K-*NFK*-F

Regardless of the precise mechanism, we can conclude that MPO adds dioxygen *via* a pathway involving compound III because this is the main form of MPO in systems containing catalase and superoxide (4, 46). Dioxygenase activity may rival halogenation by MPO in phagosomes since the majority of the enzyme is predicted to be present as compound III (4) as also suggested by difference spectrophotometry (46).

Hydroxylation of KWF by MPO in the presence of chloride gave the highest yield of any MPO-mediated reaction products observed in this study. Hydroxylation was enhanced in the presence of superoxide, which suggests that superoxide is required to maintain HOCl production by preventing accumulation of compound II. Thus, superoxide should drive hydroxylation in phagosomes, making it a likely tryptophan modification that occurs during phagocytosis. However, it is important to note that HOCl reacts 1000 times faster with other moieties including thiols and methionine residues present in the phagosome (57, 58). Consequently, HOCl-dependent oxidation of tryptophan residues would not occur in phagosomes until after the more reactive amino acids are depleted (12). In contrast, HOCl-independent dioxygenation by MPO in concert with superoxide might be a more favorable route for tryptophan oxidation during the early period of phagocytosis.

Our proteomic analysis indicates that tryptophan dioxygenation occurs during neutrophil phagocytosis of *S. aureus*. However, dioxygenated tryptophan residues were consistently detected only on peptides from calprotectin, lactoferrin, and MPO. Calprotectin is the most abundant protein in neutrophils and present in the phagosome (53), where oxidation renders it susceptible to digestion with neutrophil proteases (59). Our data suggest that the predominant route for a peptide to become dioxygenated by MPO is by binding to the enzyme’s active site, making proteolysis of proteins to small peptides that fit into this site a prerequisite for dioxygenation. Consequently, the high abundance of calprotectin and its ready proteolysis after oxidation make its tryptophan residues likely substrates for dioxygenation. In contrast, lactoferrin undergoes limited proteolysis by neutrophil proteases (60, 61) and does not associate with MPO (62). Consequently, at this time, it is not apparent why dioxygenation of its tryptophan residues is clearly observed compared with those in other neutrophil proteins.

The limited number of dioxygenated peptides consistently detected in our proteomics data does not exclude the existence of other dioxygenation targets besides calprotectin, lactoferrin, and MPO. Evaluating tryptophan dioxygenation inside the neutrophil phagosome presents a significant analytical challenge. This is because only a fraction of a given tryptophan residue becomes modified, and extensive proteolysis by neutrophil proteases during phagocytosis leads to signal dilution across numerous peptides and complicates the analysis. Therefore, while our proteomics results support MPO dioxygenation activity in the neutrophil phagosome, they cannot be used to infer the extent of tryptophan dioxygenation or identify all targets. This may be particularly relevant for bacterial peptides.

In future work, to allow quantification of tryptophan dioxygenation in the phagosome, specific antibodies and Western blotting could be used. Alternatively, phagosomal proteins could be broken down into individual amino acids followed by measurement of tryptophan hydroperoxide or NFK relative to tryptophan. Analogous approaches are used to measure 3-chlorotyrosine levels on proteins (63, 64). While the lack of quantification of tryptophan dioxygenation in a more physiological setting leaves room for further investigations, overcoming these analytical challenges will be essential to further validate the physiological relevance of the dioxygenation reaction.

Dioxygenation of tryptophan residues of phagosome constituents and their breakdown products is likely to modulate their function as antimicrobials. For example, α-defensins 1 to 3 are antimicrobial peptides with a conserved Trp-26 residue that is integral for function (65). Dioxygenation of peptides in the phagosome may also result in a gain of function for instance as chemoattractant and signaling molecules. A recent study demonstrating the ability of a tryptophan-derived hydroperoxide to regulate vascular tone and blood pressure during inflammation lends support to this proposal (35). Furthermore, products of oxidative tryptophan catabolism can induce neutrophil apoptosis (66) and inhibit neutrophil chemotaxis (67), suggesting that MPO-mediated tryptophan metabolism may function to signal resolution of inflammation. Our data suggest that MPO is capable of dioxygenating itself, which may act as a regulatory mechanism to dampen MPO activity. Based on the new discoveries made in this work, future studies should focus on whether dioxygenation occurs exclusively within phagosomes before peptides are released extracellularly or extracellular MPO dioxygenates peptides released by neutrophils. It will also be of interest to determine whether peptides are substrates for this activity of MPO inside phagosomes or, given the high concentrations of proteins within these vesicles, MPO associates with particular proteins, such as calprotectin and lactoferrin. With this knowledge and good quantitative techniques, it will be imperative to determine the extent of tryptophan oxidation that occurs in the neutrophil phagosome, and whether this plays a role in pathogen killing or immune signaling.

In summary, we have discovered a previously unrecognized tryptophan dioxygenase activity of MPO as well as demonstrating that superoxide adds to tryptophan radicals formed by the enzyme in its classic peroxidase cycle. The antimicrobial activity of MPO has hitherto been exclusively ascribed to its capacity to generate HOCl from hydrogen peroxide and chloride. The alternative activity of MPO reported here for the first time may have implications for bacterial killing by neutrophils. Analogous to the function of recognized tryptophan-metabolizing enzymes such as indolamine 2,3-dioxygenase, tryptophan oxidation by MPO may play a signaling role during infection and inflammation. Our findings demonstrate that superoxide is not simply a precursor of hydrogen peroxide but gives rise to other oxidants and oxidation products that may contribute to the function of neutrophils in infection and inflammation.

## Experimental procedures

### Materials

Catalase from bovine liver, diethylenetriaminepentaacetic acid (DTPA), l-histidine, l-methionine, tryptamine hydrochloride, l-tryptophan, cytochrome *c* from equine heart, SOD from bovine erythrocytes (Cu/Zn SOD1), 3,3′,5,5′-tetramethylbenzidine, taurine, PMA, DPI, acetaldehyde, Tris(2-carboxyethyl)phosphine), and XO (grade I, ammonium sulfate suspension) from bovine milk were purchased from Sigma. Human MPO and iodoacetamide were from Planta Natural Products and GE Healthcare, respectively. The 2-thioxanthine inhibitor AZM198 was a gift from AstraZeneca (68). HOCl was a commercial chlorine bleach product sold by Pental, and its concentration was determined spectrophotometrically using ε_292_ = 350 M^−1^ cm^−1^ at pH 12 (69). Peptides KWF and KWF-NH_2_ were obtained from Genscript. Sequencing grade trypsin (0.5 μg/μl) was from Promega. Peptides Ac-WK-NH_2_, Ac-WS-NH_2_, Ac-WV-NH_2_, Ac-WA-NH_2_, Ac-WF-NH_2_, Ac-WG-NH_2_, WF-NH_2_, WV-NH_2_, and K-*NFK*-F were synthesized by Fmoc-strategy solid-phase peptide synthesis as described in the Supporting information using standard Fmoc-protected amino acids, coupling reagents, and resins purchased from Novabiochem, Mimotopes, or GL Biochem. Peptide synthesis grade *N,N*-dimethylformamide was obtained from Labscan. For synthesis of peptide K-*NFK*-F, the NFK building block was first generated by exposing Fmoc-Trp(Boc)-OH to ozone and dimethyl sulfide as described in the Supporting information and then used for standard solid-phase peptide synthesis. Prior to experiments, peptides were dissolved at 2 mM in pH 5.0 water. For isolation of neutrophils, PBS, Hank’s buffered salt solution (HBSS), and distilled water from Gibco; dextran (from *Leuconostoc mesenteroides*, molecular weight 200,000–300,000) from MP Biomedicals; sodium chloride (5 M) and Ficoll–Paque (Cytiva; average molecular weight 150,000) from Sigma were used.

### Human peripheral neutrophils

Blood was obtained from healthy human volunteers with informed consent and ethical approval from the Southern Health & Disability Ethics Committee, New Zealand. Our studies abide by the Declaration of Helsinki principles. Neutrophils were isolated from freshly drawn heparinized blood under sterile conditions by dextran sedimentation followed by Ficoll/Hypaque centrifugation. Human granulocytes including neutrophils were isolated from the Ficoll pellet by erythrocyte lysis in hypotonic buffer (70). Neutrophils were resuspended in HBSS supplemented with 10% autologous serum. Purity (>96%) was verified using flow cytometry analysis using the characteristic forward/side scatter.

### Reaction mixtures containing KWF and PMA-stimulated peripheral neutrophils

KWF (200 μM) was mixed with neutrophils (1 × 10^6^ cells/ml) in the presence or the absence of MPO (100 nM) and SOD (20 μg/ml). PMA (100 ng/ml) was added to stimulate the neutrophils, and reaction mixtures were incubated for 30 min at 37 °C with end-over-end rotation. Under these conditions, neutrophils will produce on average 8 μM O_2_^.−^/min (40). Neutrophils were removed by centrifugation at 100*g*, and the supernatant was injected for LC–MS analysis described later.

### Reaction mixtures containing MPO and superoxide generated by XO

A superoxide flux was generated using the XO/acetaldehyde system (71). The commercial suspension of XO was diluted 1:50 in 100 mM phosphate buffer (pH 7.4) with 10 μM DTPA prior to adding to reaction mixtures containing peptides and MPO. The rate of superoxide generated by the diluted XO was determined by monitoring the reduction of cytochrome *c*. Cytochrome *c* (0.24 mg/ml), acetaldehyde (10 mM), and catalase (20 μg/ml) were mixed in 100 mM phosphate buffer (pH 7.4) with 10 μM DTPA, XO (1:50 dilution) was added, and the absorbance at 550 nm was measured every 15 s for 5 min at 37 °C at a slit width of 0.5 nm using a Hitachi U-3900 spectrophotometer. The rate of absorbance change was determined and converted to the rate of superoxide production using ϵ_550_ = 21.1 × 10^3^ M^−1^ cm^−1^.

To investigate dioxygenation of tryptophan on peptides, reaction mixtures contained 100 μM of the peptide of interest, tryptophan or tryptamine, acetaldehyde (10 mM), MPO (100 nM) in 100 mM phosphate, pH 7.4 with 10 μM DTPA. For some experiments, SOD (20 μg/ml), the MPO inhibitor AZM198 (40 μM), catalase (20 μg/ml), sodium chloride (100 mM), methionine (1 mM), or histidine (1 mM) were included. The reaction was started by the addition of XO (4 μM O_2_^.-^/min or increasing concentrations) and allowed to proceed for 30 min at 22 to 24 °C. Because our experiments were done at 22 to 24 °C, but our cytochrome *c* assays were performed at 37 °C, we retrospectively adjusted our measured rates by 50%. This adjustment was based on a separate calibration measurement showing that the rate of superoxide production at 22 to 24 °C is half the rate at 37 °C. Reactions were stopped by adding SOD (20 μg/ml).

### Phagocytosis of opsonized *S. aureus* by neutrophils

The *S. aureus* 502a strain (American Type Culture Collection 27217) was stored and grown under standard conditions and maintained on tryptic soy broth blood agar plates. Prior to experiments, 10 ml of lysogeny broth was inoculated with a single colony of *S. aureus* and grown overnight in a shaking incubator at 37 °C at 200 rpm. Bacteria were pelleted by centrifugation at 10,000*g* for 3 min and washed twice with PBS and then resuspended in HBSS. Bacterial aggregates were removed by a slow spin at 100*g* for 5 min, and the concentration of bacteria in the supernatant was estimated by absorbance measurement, where an absorbance of 0.13 at 550 nm corresponded to approximately 1 × 10^8^ bacteria/ml. Bacteria were opsonized at estimated 2 × 10^8^/ml with 10% serum pooled from six healthy donors for 20 min at 37 °C with end-over-end rotation. Bacteria were pelleted at 10,000*g* for 6 min and resuspended in the same volume of HBSS without serum.

Neutrophils were isolated as described previously and incubated at 2 × 10^7^ cells/ml in HBSS with 20 μM of the MPO inhibitor AZM198 or the NADPH oxidase inhibitor DPI at 37 °C for 10 min. Both the opsonized bacteria and preincubated neutrophils were prepared at twice their final concentration and were mixed together in equal volume for the phagocytosis incubation with final concentrations of 10^7^ cells/ml neutrophils and a ratio of approximately 10:1 bacteria:neutrophil, with or without inhibitors present at 10 μM. A sample of opsonized bacteria was plated onto agar to determine the multiplicity of infection by colony counting. The average multiplicity of infection was 14 ± 3 (mean ± SD). All tubes, including a neutrophils-only control (unstim) at 10^7^/ml in HBSS, were incubated at 37° C with end-over-end rotation for 2 h. Neutrophils were pelleted by centrifugation in a swing-out rotor at 100*g* for 5 min at 4 °C. The supernatant was kept as the extracellular fraction. Neutrophils were lysed by resuspension in 1 ml (pH 11) water for 5 min at room temperature including eight strokes of passage through a 25G syringe needle. Cell debris was removed by centrifugation at 30,000*g* for 20 min at 4 °C. The clarified lysates and the extracellular fractions were evaporated to dryness in a SpeedVac vacuum concentrator before processing for Orbitrap MS analysis.

### LC–MS/MS analyses of purified peptides, tryptophan or tryptamine

Reaction mixtures containing purified peptides, tryptophan or tryptamine, were analyzed using an UltiMate 3000 HPLC system coupled inline to a Velos Pro mass spectrometer (Thermo Fisher Scientific). Ten microliters of the reaction mixtures containing peptides was injected onto a Jupiter Proteo 90 Å column (4 μm, 150 mm × 2 mm; Phenomenex). For analysis of tryptamine and tryptophan-containing reaction mixtures, an Acclaim Polar Advantage 120 Å column (2.2 μm, 150 × 2.1 mm; Thermo Fisher Scientific) was used and 10 and 25 μl were injected, respectively. After operating the columns isocratically at 98% solvent A (0.1% formic acid in water)/2% solvent B (0.1% formic acid in acetonitrile) for 3 min, an acetonitrile gradient was run to 30% solvent B over 18 min. Solvent B was held at 100% for 5 min followed by column re-equilibration for 5 min with 98% solvent A. The flow rate was 250 μl/min, and the column temperature was 60 °C. The spray voltage was 4 kV, the source and capillary temperature 400 and 275 °C, respectively, and sheath gas flow 15 AU. Collision-induced dissociation–MS/MS spectra in positive ion mode were acquired for the 10 most abundant precursors with a signal intensity of greater than 500. Isolation width was 1 *m*/z, the normalized collision energy was 35 AU, and activation time was 10 ms. The same fragmentation parameters were applied for MS^3^ experiments. Ions of interest were extracted, and their resulting peak area (area under the curve [AUC]) was determined using Thermo XCalibur 4.2.47 Qual Browser.

### Orbitrap LC–MS/MS analyses of neutrophil lysates

Dried neutrophil lysates and extracellular fractions were solubilized in 0.8 ml of 100 mM triethylammonium bicarbonate by repeated vortex and sonication until the samples were visibly dissolved. Following centrifugation at 30,000*g* for 30 min, supernatants were incubated with 5 mM Tris(2-carboxyethyl)phosphine and 10 mM iodoacetamide in the dark at 20 to 22 °C for 15 min. Samples were filtered using 3 kDa cutoff Amicon ultracentrifugal filters and four exchanges of triethylammonium bicarbonate. The protein concentration was determined by Bradford assay according to the manufacturer’s specification (Bio-Rad), and trypsin was added at a ratio of 1:25 and incubated overnight at 37 °C. Tryptic digests were dried in a vacuum evaporator, then resolubilized in 30 μl of 5% (v/v) acetonitrile, 0.1% (v/v) formic acid by vortexing and sonication.

Samples were analyzed on an Orbitrap Exploris 240 mass spectrometer (Thermo Fisher Scientific) coupled to a Vanquish Neo nanoflow uHPLC system (Thermo Fisher Scientific). Injections were normalized to an 8 μg protein equivalent volume based on the total ion count derived from a test MS measurement and an in-house standard curve. Each sample was injected in triplicate. Samples were separated on 75 μm ID silica emitter tip capillary column of 25 cm in length (CoAnn Technology), which was packed inhouse with Luna C18 3 μm bead material (Phenomenex). Starting conditions were 95% solvent A (0.1% formic acid in water)/5% solvent B (0.1% formic acid in acetonitrile) at a flow rate of 0.4 μl/min, then a stepwise acetonitrile gradient was run from 5 to 25% over 100 min, 25 to 45% over 8 min, and 45% to 98% B over 6 min. MS1 was scanned from 300 to 2000 *m/z* range, and the +1 and higher charge state MS1 ions were allowed for MS2 fragmentation. The higher energy collisional dissociation stepped energy was set at 28, 32, and 35. The dynamic exclusion was enabled for one repeat count with an exclusion duration of 18 s. The total cycle time was set at 2.5 s.

To identify proteins and peptides, database searches were performed in Proteome Discoverer 2.5.0.4 (Thermo Fisher Scientific) using the embedded Sequest HT search engine. Triplicate measurements for each sample were processed together in the same workflow within Proteome Discoverer. The following processing workflow settings were applied: precursor mass tolerance, 10 ppm; fragment mass tolerance, 0.02 Da; maximum number of missed cleavages, 2; minimum peptide length: 4, UniProt database, *Homo sapiens* and *S. aureus*; dynamic modification, oxidation (+15.995 Da, M, W), dioxidation (+31.990 Da, C, M, W), trioxidation (+47.985 Da, C), carbamidomethyl (+57.021 Da, C); cleavage reagents, trypsin (K, R). Percolator was integrated into our workflow to improve the confidence of peptide and protein identifications and to reduce the number of false positives. The minimum number of peptide sequences per protein was 1. The false discovery rate was set to 1 and 5% for peptide or protein hits with high and medium confidence, respectively.

The extracted ion chromatogram for the *m/z* of selected dioxygenated peptides, that is, LTW(O_2_)ASHEK and GADVW(O_2_)FK, in neutrophil lysates, and EDAIW(O_2_)NLLR in extracellular samples, was generated using Thermo XCalibur 4.2.47 Qual Browser, with a 5-ppm mass tolerance and a mass precision of four decimals. The AUC for the peaks associated with the highest-scoring peptide spectrum match in the control sample was determined through manual integration. The AUC ratio for the dioxygenated peptide relative to each of the nonoxidized peptides (LTWASHEK, GADVWFK, and EDAIWNLLR) was calculated for each of the triplicate measurements. The mean of these AUC ratios was then determined by averaging the individual ratios across the three replicates.

### Pulse radiolysis experiments

Pulse radiolysis was performed using the Dynaray 4 MV linear accelerator facility at the University of Auckland, New Zealand, which has an adjunct computer-controlled optical detection system fitted with Supracil optics (72). Electron pulses of 200 ns to 2 μs duration were used to deliver radiolytic doses of 19 to 53 Gy (J/kg) to the temperature-controlled reaction cell (set at 25 °C), as determined by using the standard potassium thiocyanate dosimeter to calibrate a surrounding charge collection plate to correct for small variations in the delivered doses (73). Pulse radiolysis was carried out in O_2_-saturated solutions containing 100 mM formate ions, which convert the radiolytic hydroxyl radicals and H-atoms to superoxide *via* the carbon dioxide radical anion, CO_2_^.-^. The combined concentration of superoxide produced in O_2_-saturated solutions, from the e_aq_^-^, hydroxyl radicals, and H-atoms is taken as 0.64 μM Gy^−1^(μmol.J^−1^). The solutions also contained 0.72 μM MPO (per heme) in 25 mM phosphate (pH 7.4), with 20 μM DTPA, plus or minus 200 μM KWF. Reaction of superoxide with MPO was monitored at 430 nm, the maximum of the Soret peak for ferric enzyme, and 452 or 625 nm; the Soret and visible peaks for compound III. First-order rate constants were determined from exponential fits to the change in absorbance over time.

### Statistical analysis

Data were analyzed and graphed using GraphPad Prism Software, version 8.2.1 (GraphPad Software, Inc). Statistical significance was determined using tests described in the figure legends, and a *p* value of less than 0.05 was considered significant.

## Data availability

The authors confirm that the data supporting the findings of this study are available within the article and its supporting information.

## Supporting information

This article contains supporting information (74).

## Conflict of interest

The authors declare that they have no conflicts of interest with the contents of this article.

## Supporting information

Supporting Information

Exp1 Extracellular

Exp1 Lysate

Exp2 Extracellular

Exp2 Lysate

Exp3 Extracellular

Exp3 Lysate

## References

[1] Kettle A.J., Ashby L.V., Winterbourn C.C., Dickerhof N. (2023). Superoxide: the enigmatic chemical chameleon in neutrophil biology. Immunol. Rev..

[2] Gebicki J.M., Bielski B.H.J. (1981). Comparison of the capacities of the perhydroxyl and the superoxide radicals to initiate chain oxidation of linoleic-acid. J. Am. Chem. Soc..

[3] Imlay J.A. (2013). The molecular mechanisms and physiological consequences of oxidative stress: lessons from a model bacterium. Nat. Rev. Microbiol..

[4] Kettle A.J., Anderson R.F., Hampton M.B., Winterbourn C.C. (2007). Reactions of superoxide with myeloperoxidase. Biochemistry.

[5] Schultz J., Kaminker K. (1962). Myeloperoxidase of the leucocyte of normal human blood. I. Content and localization. Arch. Biochem. Biophys..

[6] Harrison J.E., Schultz J. (1976). Studies on the chlorinating activity of myeloperoxidase. J. Biol. Chem..

[7] Winterbourn C.C., Kettle A.J., Hampton M.B. (2016). Reactive oxygen species and neutrophil function. Annu. Rev. Biochem..

[8] Chapman A.L., Hampton M.B., Senthilmohan R., Winterbourn C.C., Kettle A.J. (2002). Chlorination of bacterial and neutrophil proteins during phagocytosis and killing of *Staphylococcus aureus*. J. Biol. Chem..

[9] Green J.N., Kettle A.J., Winterbourn C.C. (2014). Protein chlorination in neutrophil phagosomes and correlation with bacterial killing. Free Radic. Biol. Med..

[10] Ashby L.V., Springer R., Loi V.V., Antelmann H., Hampton M.B., Kettle A.J. (2022). Oxidation of bacillithiol during killing of *Staphylococcus aureus* USA300 inside neutrophil phagosomes. J. Leukoc. Biol..

[11] Parker H.A., Dickerhof N., Forrester L., Ryburn H., Smyth L., Messens J. (2021). Mycobacterium smegmatis resists the bactericidal activity of hypochlorous acid produced in neutrophil phagosomes. J. Immunol..

[12] Winterbourn C.C., Hampton M.B., Livesey J.H., Kettle A.J. (2006). Modeling the reactions of superoxide and myeloperoxidase in the neutrophil phagosome: implications for microbial killing. J. Biol. Chem..

[13] Albrett A.M., Ashby L.V., Dickerhof N., Kettle A.J., Winterbourn C.C. (2018). Heterogeneity of hypochlorous acid production in individual neutrophil phagosomes revealed by a rhodamine-based probe. J. Biol. Chem..

[14] Beaman B.L., Black C.M., Doughty F., Beaman L. (1985). Role of superoxide dismutase and catalase as determinants of pathogenicity of Nocardia asteroides: importance in resistance to microbicidal activities of human polymorphonuclear neutrophils. Infect. Immun..

[15] Hampton M.B., Kettle A.J., Winterbourn C.C. (1996). Involvement of superoxide and myeloperoxidase in oxygen-dependent killing of Staphylococcus aureus by neutrophils. Infect. Immun..

[16] Johnston R.B., Keele B.B., Misra H.P., Lehmeyer J.E., Webb L.S., Baehner R.L. (1975). The role of superoxide anion generation in phagocytic bactericidal activity. Studies with normal and chronic granulomatous disease leukocytes. J. Clin. Invest..

[17] McDonald R.J., Berger E.M., White C.W., White J.G., Freeman B.A., Repine J.E. (1985). Effect of superoxide dismutase encapsulated in liposomes or conjugated with polyethylene glycol on neutrophil bactericidal activity in vitro and bacterial clearance in vivo. Am. Rev. Respir. Dis..

[18] Ximenes V.F., Silva S.O., Rodrigues M.R., Catalani L.H., Maghzal G.J., Kettle A.J. (2005). Superoxide-dependent oxidation of melatonin by myeloperoxidase. J. Biol. Chem..

[19] Carroll L., Pattison D.I., Davies J.B., Anderson R.F., Lopez-Alarcon C., Davies M.J. (2018). Superoxide radicals react with peptide-derived tryptophan radicals with very high rate constants to give hydroperoxides as major products. Free Radic. Biol. Med..

[20] Jin F.M., Leitich J., Vonsonntag C. (1993). The superoxide radical reacts with tyrosine-derived phenoxyl radicals by addition rather than by electron-transfer. J. Chem. Soc. Perk T.

[21] Meotti F.C., Jameson G.N., Turner R., Harwood D.T., Stockwell S., Rees M.D. (2011). Urate as a physiological substrate for myeloperoxidase: implications for hyperuricemia and inflammation. J. Biol. Chem..

[22] Winterbourn C.C., Kettle A.J. (2003). Radical-radical reactions of superoxide: a potential route to toxicity. Biochem. Biophys. Res. Commun..

[23] Koppenol W.H., Stanbury D.M., Bounds P.L. (2010). Electrode potentials of partially reduced oxygen species, from dioxygen to water. Free Radic. Biol. Med..

[24] Furtmuller P.G., Arnhold J., Jantschko W., Pichler H., Obinger C. (2003). Redox properties of the couples compound I/compound II and compound II/native enzyme of human myeloperoxidase. Biochem. Biophys. Res. Commun..

[25] Boreen A.L., Edhlund B.L., Cotner J.B., McNeill K. (2008). Indirect photodegradation of dissolved free amino acids: the contribution of singlet oxygen and the differential reactivity of DOM from various sources. Environ. Sci. Technol..

[26] Baptista M.S., Cadet J., Greer A., Thomas A.H. (2021). Photosensitization reactions of biomolecules: definition, targets and mechanisms. Photochem. Photobiol..

[27] Dahl T.A., Midden W.R., Hartman P.E. (1989). Comparison of killing of gram-negative and gram-positive bacteria by pure singlet oxygen. J. Bacteriol..

[28] Steinbeck M.J., Khan A.U., Karnovsky M.J. (1992). Intracellular singlet oxygen generation by phagocytosing neutrophils in response to particles coated with a chemical trap. J. Biol. Chem..

[29] Efimov I., Basran J., Thackray S.J., Handa S., Mowat C.G., Raven E.L. (2011). Structure and reaction mechanism in the heme dioxygenases. Biochemistry.

[30] Marquez L.A., Dunford H.B. (1990). Reaction of compound III of myeloperoxidase with ascorbic acid. J. Biol. Chem..

[31] Ximenes V.F., Maghzal G.J., Turner R., Kato Y., Winterbourn C.C., Kettle A.J. (2009). Serotonin as a physiological substrate for myeloperoxidase and its superoxide-dependent oxidation to cytotoxic tryptamine-4,5-dione. Biochem. J..

[32] Kettle A.J., Winterbourn C.C. (1994). Superoxide-dependent hydroxylation by myeloperoxidase. J. Biol. Chem..

[33] Ehrenshaft M., Deterding L.J., Mason R.P. (2015). Tripping up Trp: modification of protein tryptophan residues by reactive oxygen species, modes of detection, and biological consequences. Free Radic. Biol. Med..

[34] Ronsein G.E., de Oliveira M.C., de Medeiros M.H., Di Mascio P. (2009). Characterization of O(2) ((1)delta(g))-derived oxidation products of tryptophan: a combination of tandem mass spectrometry analyses and isotopic labeling studies. J. Am. Soc. Mass Spectrom..

[35] Stanley C.P., Maghzal G.J., Ayer A., Talib J., Giltrap A.M., Shengule S. (2019). Singlet molecular oxygen regulates vascular tone and blood pressure in inflammation. Nature.

[36] Kettle A.J., Candaeis L.P. (2000). Oxidation of tryptophan by redox intermediates of myeloperoxidase and inhibition of hypochlorous acid production. Redox Rep..

[37] Jantschko W., Furtmuller P.G., Allegra M., Livrea M.A., Jakopitsch C., Regelsberger G. (2002). Redox intermediates of plant and mammalian peroxidases: a comparative transient-kinetic study of their reactivity toward indole derivatives. Arch. Biochem. Biophys..

[38] Burner U., Jantschko W., Obinger C. (1999). Kinetics of oxidation of aliphatic and aromatic thiols by myeloperoxidase compounds I and II. FEBS Lett..

[39] Hori H., Fenna R.E., Kimura S., Ikeda-Saito M. (1994). Aromatic substrate molecules bind at the distal heme pocket of myeloperoxidase. J. Biol. Chem..

[40] Schindler L., Smyth L.C.D., Bernhagen J., Hampton M.B., Dickerhof N. (2021). Macrophage migration inhibitory factor (MIF) enhances hypochlorous acid production in phagocytic neutrophils. Redox Biol..

[41] Matheson I.B.C., Lee J. (1979). Chemical-reaction rates of amino-acids with singlet oxygen. Photochem. Photobiol..

[42] Caldwell K.A., Tappel A.L. (1964). Reactions of seleno- and sulfoamino acids with hydroperoxides. Biochemistry.

[43] Nakagawa M., Kato S., Kataoka S., Kodato S., Watanabe H., Okajima H. (1981). Dye-sensitized photo-oxygenation of tryptophan - 3a-hydroperoxypyrroloindole as a labile precursor of formylkynurenine. Chem. Pharm. Bull..

[44] Gracanin M., Hawkins C.L., Pattison D.I., Davies M.J. (2009). Singlet-oxygen-mediated amino acid and protein oxidation: formation of tryptophan peroxides and decomposition products. Free Radic. Biol. Med..

[45] Lam H.Y., Zhang Y., Liu H., Xu J., Wong C.T., Xu C. (2013). Total synthesis of daptomycin by cyclization via a chemoselective serine ligation. J. Am. Chem. Soc..

[46] Winterbourn C.C., Garcia R.C., Segal A.W. (1985). Production of the superoxide adduct of myeloperoxidase (compound III) by stimulated human neutrophils and its reactivity with hydrogen peroxide and chloride. Biochem. J..

[47] Kettle A.J., Sangster D.F., Gebicki J.M., Winterbourn C.C. (1988). A pulse radiolysis investigation of the reactions of myeloperoxidase with superoxide and hydrogen peroxide. Biochim. Biophys. Acta.

[48] Dellegar S.M., Murphy S.A., Bourne A.E., DiCesare J.C., Purser G.H. (1999). Identification of the factors affecting the rate of deactivation of hypochlorous acid by melatonin. Biochem. Biophys. Res. Commun..

[49] Hawkins C.L. (2020). Hypochlorous acid-mediated modification of proteins and its consequences. Essays Biochem..

[50] Peng D.Q., Brubaker G., Wu Z., Zheng L., Willard B., Kinter M. (2008). Apolipoprotein A-I tryptophan substitution leads to resistance to myeloperoxidase-mediated loss of function. Arterioscler. Thromb. Vasc. Biol..

[51] Maallem H., Sheppard K., Fletcher J. (1982). The discharge of primary and secondary granules during immune phagocytosis by normal and chronic granulocytic leukaemia polymorphonuclear neutrophils. Br. J. Haematol..

[52] Leffell M.S., Spitznagel J.K. (1975). Fate of human lactoferrin and myeloperoxidase in phagocytizing human neutrophils: effects of immunoglobulin G subclasses and immune complexes coated on latex beads. Infect. Immun..

[53] Burlak C., Whitney A.R., Mead D.J., Hackstadt T., Deleo F.R. (2006). Maturation of human neutrophil phagosomes includes incorporation of molecular chaperones and endoplasmic reticulum quality control machinery. Mol. Cell Proteomics.

[54] Molloy A.L., Winterbourn C.C. (1990). Release of iron from phagocytosed Escherichia coli and uptake by neutrophil lactoferrin. Blood.

[55] Bielski B.H.J., Allen A.O. (1977). Mechanism of disproportionation of superoxide radicals. J. Phys. Chem..

[56] Winterbourn C.C. (1986). Myeloperoxidase as an effective inhibitor of hydroxyl radical production. Implications for the oxidative reactions of neutrophils. J. Clin. Invest..

[57] Pattison D.I., Davies M.J. (2001). Absolute rate constants for the reaction of hypochlorous acid with protein side chains and peptide bonds. Chem. Res. Toxicol..

[58] Storkey C., Davies M.J., Pattison D.I. (2014). Reevaluation of the rate constants for the reaction of hypochlorous acid (HOCl) with cysteine, methionine, and peptide derivatives using a new competition kinetic approach. Free Radic. Biol. Med..

[59] Hoskin T.S., Crowther J.M., Cheung J., Epton M.J., Sly P.D., Elder P.A. (2019). Oxidative cross-linking of calprotectin occurs in vivo, altering its structure and susceptibility to proteolysis. Redox Biol..

[60] Britigan B.E., Edeker B.L. (1991). Pseudomonas and neutrophil products modify transferrin and lactoferrin to create conditions that favor hydroxyl radical formation. J. Clin. Invest..

[61] Britigan B.E., Hayek M.B., Doebbeling B.N., Fick R.B. (1993). Transferrin and lactoferrin undergo proteolytic cleavage in the Pseudomonas aeruginosa-infected lungs of patients with cystic fibrosis. Infect. Immun..

[62] Sokolov A.V., Zakharova E.T., Kostevich V.A., Samygina V.R., Vasilyev V.B. (2014). Lactoferrin, myeloperoxidase, and ceruloplasmin: complementary gearwheels cranking physiological and pathological processes. Biometals.

[63] Hazen S.L., Crowley J.R., Mueller D.M., Heinecke J.W. (1997). Mass spectrometric quantification of 3-chlorotyrosine in human tissues with attomole sensitivity: a sensitive and specific marker for myeloperoxidase-catalyzed chlorination at sites of inflammation. Free Radic. Biol. Med..

[64] Kettle A.J., Chan T., Osberg I., Senthilmohan R., Chapman A.L., Mocatta T.J. (2004). Myeloperoxidase and protein oxidation in the airways of young children with cystic fibrosis. Am. J. Respir. Crit. Care Med..

[65] Wei G., Pazgier M., de Leeuw E., Rajabi M., Li J., Zou G. (2010). Trp-26 imparts functional versatility to human alpha-defensin HNP1. J. Biol. Chem..

[66] El-Zaatari M., Chang Y.M., Zhang M., Franz M., Shreiner A., McDermott A.J. (2014). Tryptophan catabolism restricts IFN-gamma-expressing neutrophils and Clostridium difficile immunopathology. J. Immunol..

[67] Loughman J.A., Yarbrough M.L., Tiemann K.M., Hunstad D.A. (2016). Local generation of kynurenines mediates inhibition of neutrophil chemotaxis by uropathogenic Escherichia coli. Infect. Immun..

[68] Tiden A.K., Sjogren T., Svensson M., Bernlind A., Senthilmohan R., Auchere F. (2011). 2-Thioxanthines are mechanism-based inactivators of myeloperoxidase that block oxidative stress during inflammation. J. Biol. Chem..

[69] Morris J.C. (1966). Acid ionization constant of HOCl from 5 to 35 degrees. J. Phys. Chem..

[70] Magon N.J., Parker H.A., Ashby L.V., Springer R.J., Hampton M.B., Quinn M.T., DeLeo F.R. (2020). Neutrophil: Methods and Protocols.

[71] Dix T.A., Hess K.M., Medina M.A., Sullivan R.W., Tilly S.L., Webb T.L. (1996). Mechanism of site-selective DNA nicking by the hydrodioxyl (perhydroxyl) radical. Biochemistry.

[72] Anderson R.F., Denny W.A., Li W.J., Packer J.E., Tercel M., Wilson W.R. (1997). Pulse radiolysis studies on the fragmentation of arylmethyl quaternary nitrogen mustards by one-electron reduction in aqueous solution. J. Phys. Chem. A..

[73] Schuler R.H., Patterson L.K., Janata E. (1980). Yield for the scavenging of Oh radicals in the radiolysis of N2o-saturated aqueous-solutions. J. Phys. Chem..

[74] Dypbukt J.M., Bishop C., Brooks W.M., Thong B., Eriksson H., Kettle A.J. (2005). A sensitive and selective assay for chloramine production by myeloperoxidase. Free Radic. Biol. Med..

