# Supplementary material for: Dioxygenation of tryptophan residues by superoxide and myeloperoxidase
Source: J Biol Chem. 2025 Mar 11;301(4):108402. doi: 10.1016/j.jbc.2025.108402 (PMC12017991; doi:10.1016/j.jbc.2025.108402)
Supplement: Supporting Information [file mmc1.pdf]

Supporting Information for: Dioxygenation of tryptophan residues by superoxide and myeloperoxidase

Nina Dickerhof, Louisa V Ashby, Daniel Ford, Joshua J Dilly, Robert F Anderson, Richard J Payne, Anthony J Kettle

Contents

Supplementary Experimental Procedures for Peptide Synthesis 2
General Procedures 2
General Procedures for Solid-phase Peptide Synthesis (SPPS) 2
Synthesis of Peptides 5
Mass spectra of peptides 11
Supporting Data 16

Supplementary Experimental Procedures for Peptide Synthesis

General Procedures

UPLC-MS was performed on a Shimadzu LC-MS 2020 system with a Nexera X2 LC-30AD pump and a Nexera X2 SPD-M30A diode array detector coupled to a Shimadzu 2020 mass spectrometer (ESI) operating in positive mode. Peptides were analyzed using an Acquity UPLC BEH 1.7 µm (C18) 2.1 x 50 mm column at a flow rate of 0.6 mL min^{-1} using a mobile phase of water with 0.1 vol.% TFA and acetonitrile with 0.1 vol.% TFA. Preparative reversed-phase HPLC was performed using a Waters 600 Multisolvent Delivery System and Waters 500 pump with 2996 photodiode array detector or Waters 490E Programmable Wavelength Detector operating at 210 and 280 nm. All peptides were purified with a Waters Sunfire 5 µm (C18; 19 x 150 mm) preparative column operating at a flow rate of 14 mL min^{-1} using a mobile phase of water with 0.1 vol.% TFA and acetonitrile with 0.1 vol.% TFA with a linear gradient as specified. Low-resolution mass spectra were obtained on a Shimadzu 2020 mass spectrometer (ESI) operating in positive mode.

General Procedures for Solid-phase Peptide Synthesis (SPPS)

Reagents and Solvents

Peptide-grade *N,N*-dimethylfomamide (DMF) and dichloromethane (DCM) were purchased from RCI Labscan and Merck, respectively. Acetonitrile (MeCN) for chromatography was purchased as ‘gradient grade’ from Sigma-Aldrich and ultrapure water was from a Merck Millipore Direct-Q 5 water purification system. All solvents for chromatography were supplemented with formic acid (FA) purchased from Sigma-Aldrich. All standard Fmoc-protected amino acids were purchased from Mimotopes. 2-chlorotrityl resin for peptide synthesis was purchased from Mimotopes.

Manual SPPS Method

Fmoc deprotection: Resin-bound peptide (0.1 mmol) was shaken in piperidine/DMF (1:4 v/v, 4 mL, 2 x 10 min). The deprotection solution was drained and the resin washed with DMF (3 x 5 mL), DCM (3 x 5 mL) and DMF (3 x 5 mL). The resultant *N*-terminal amine was then subjected to amino acid coupling conditions.

Coupling of Fmoc-protected amino acid: Resin-bound peptide (0.1 mmol) was shaken in a solution of Fmoc-amino acid (8 eq., 0.2 M), Oxyma (8 eq., 114 mg, 0.2 M) and DIC (8 eq., 125 μL, 0.2 M) in DMF (4 mL) for 2 h at room temperature. The coupling solution was drained, and the resin washed with DMF (3 x 5 mL), DCM (3 x 5 mL) and DMF (3 x 5 mL).

Capping by acetylation: After coupling, any unreacted amines were capped by acetylation. Resin-bound peptide (0.1 mmol) was shaken in Ac_2O/pyridine (1:9 v/v, 4 mL, 10 min). The capping solution was drained, and the resin washed with DMF (3 x 5 mL), DCM (3 x 5 mL) and DMF (3 x 5 mL).

Manual Fmoc-Phe-OH loading onto CTC resin: Mimotopes 2-chlorotrityl resin (0.4 mmol) was swollen by shaking in DCM/DIPEA for 30 min. The solvent was drained, and the resin washed with DMF (3 x 5 mL), DCM (3 x 5 mL) and DMF (3 x 5 mL). A coupling solution of Fmoc-Phe-OH (8 eq., 937 mg, 0.2 M), DCM (5 mL) and DIPEA (0.5 mL) was prepared and added to the resin, which was then shaken for 15 h at room temperature. The resin was washed with DMF (3 x 5 mL), DCM (3 x 5 mL) and DMF (3 x 5 mL). To estimate loading, the resin was deprotected again with piperidine/DMF (1:4 v/v, 4 mL, 2 x 10 min) and this solution retained for determination of resin loading. The resin was washed with DMF (3 x 5 mL), DCM (3 x 5 mL) and DMF (3 x 5 mL) once more before the resin was dried.

Determination of resin loading: The deprotection solution was diluted volumetrically 250-fold in piperidine/DMF (1:4 v/v). To estimate resin loading, the fulvene-piperidine adduct (ε = 7800 $M^{-1}cm^{-1}$) was quantified by UV-vis absorbance at λ = 301 nm (using a Shimadzu UV-1280 UV-vis spectrometer).

Rink Amide Resin Loading

Rink amide resin (0.6 mmol/g) was swollen for 30 min in DCM, then washed with DCM (5 x 3 mL). The resin was then treated with 20 vol.% piperidine in DMF (3 mL, 2 x 5 min) to remove the Fmoc group, then washed with DMF (5 x 3 mL), DCM (5 x 3 mL) and DMF (5 x 3 mL). A solution containing Fmoc-AA-OH (4 eq.), PyBop (4 eq.) and N-methyl-morpholine (8 eq.) in DMF (final amino acid concentration of 0.125 M) was added and the resin was shaken for 2 h at room temperature. The solution was drained and the resin washed with DMF (5 x 3 mL) and DCM (5 x 3 mL). The resin was then treated with capping solution containing 10 vol.% Ac_2O in pyridine for 10 min at room

temperature. The resin was then washed with DCM (5 x 3 mL), DMF (5 x 3 mL) and DCM (5 x 3 mL).

Coupling of Fmoc-Trp(Boc)-OH

The resin was treated with 20 vol.% piperidine in DMF (3 mL, 2 x 5 min) then washed with DMF (5 x 3 mL), DCM (5 x 3 mL) and DMF (5 x 3 mL). A solution containing amino acid (4 eq.), PyBop (4 eq.) and *N*-methyl-morpholine (8 eq.) was added to the resin and shaken at room temperature for 2 h. The resin was washed with DCM (5 x 3 mL), DMF (5 x 3 mL) and DCM (5 x 3 mL). The resin wash treated with 20 vol.% piperidine in DMF (3 mL, 2 x 5 min), then washed with DMF (5 x 3 mL) and DCM (5 x 3 mL). N-acetylated peptides were then treated with capping solution as above and washed with DCM (5 x 3 mL), DMF (5 x 3 mL) and DCM (5 x 3 mL).

Cleavage from Resin

A cleavage solution comprising TFA, tri*iso*propylsilane and water (18:1:1 v/v/v, 5 mL) was added to the resin and agitated for 2 h at room temperature. The resin was filtered and washed with DCM (2 x 3 mL). The cleavage solution and washes were combined and concentrated under nitrogen flow.

Work up and Purification

Ice-cold diethyl ether (30 mL) was added to the concentrated cleavage solution to precipitate the peptide, which was pelleted by centrifugation. The supernatant was decanted and residual diethyl ether removed under gentle nitrogen flow. After analysis by LC-MS (ESI), the crude peptide was purified by RP-HPLC.

Synthesis of Peptides

Synthesis of WF-NH_2

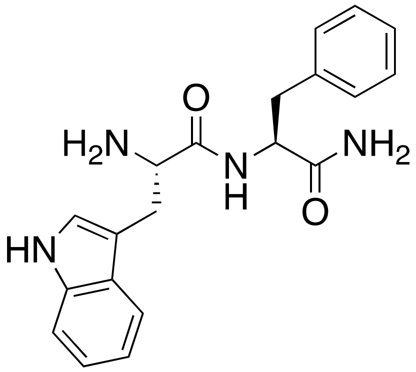

Fmoc-Phe-OH was loaded onto Rink Amide resin (100 µmol) as per the general procedures. Fmoc-Trp(Boc)-OH was then coupled for 2 h as described in the general procedures. The resin-bound peptide was treated with TFA, tri*iso*propylsilane and water (18:1:1 v/v/v, 5 mL) for cleavage from the resin and removal of protecting groups, as detailed in the general procedures. Crude peptide was purified by reversed-phase HPLC (0 to 50% B over 50 min, 0.1% TFA) to afford WF-NH_2 (13.6 mg, 39%). **LR-MS (+ESI)** m/z = 351.45 $[M+H]^+$.

Synthesis of WV-NH_2

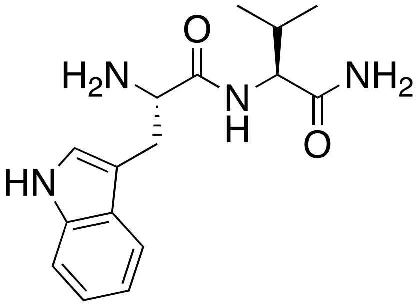

Fmoc-Val-OH was loaded onto Rink Amide resin (100 µmol) as per the general procedures. Fmoc-Trp(Boc)-OH was then coupled for 2 h as described in the general procedures. The resin-bound peptide was treated with TFA, tri*iso*propylsilane and water (18:1:1 v/v/v, 5 mL) for cleavage from the resin and removal of protecting groups, as detailed in the general procedures. Crude peptide was purified by reversed-phase

HPLC (0 to 50% B over 50 min, 0.1% TFA) to afford WV-NH_2 (10.0 mg, 33%). **LR-MS (+ESI)** m/z = 303.1 $[M+H]^+$.

Synthesis of Ac-WA-NH_2

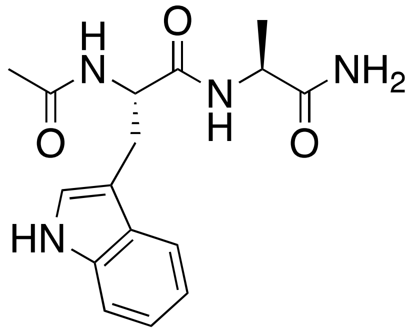

Fmoc-Ala-OH was loaded onto Rink Amide resin (100 µmol) as per the general procedures. Fmoc-Trp(Boc)-OH was then coupled for 2 h as described in the general procedures, including a final capping step for *N*-acetylation. The resin-bound peptide was treated with TFA, tri*iso*propylsilane and water (18:1:1 v/v/v, 5 mL) for cleavage from the resin and removal of protecting groups, as detailed in the general procedures. Crude peptide was purified by reversed-phase HPLC (0 to 50% B over 50 min, 0.1% TFA) to afford Ac-WA-NH_2 (17.1 mg, 54%). **LR-MS (+ESI)** m/z = 317.15 $[M+H]^+$.

Synthesis of Ac-WF-NH_2

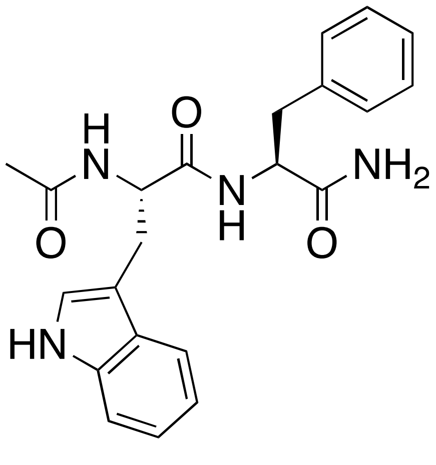

Fmoc-Phe-OH was loaded onto Rink Amide resin (100 µmol) as per the general procedures. Fmoc-Trp(Boc)-OH was then coupled for 2 h as described in the general procedures, including a final capping step for *N*-acetylation. The resin-bound peptide was treated with TFA, tri*iso*propylsilane and water (18:1:1 v/v/v, 5 mL) for cleavage from the resin and removal of protecting groups, as detailed in the general procedures. Crude peptide was purified by reversed-phase HPLC (0 to 50% B over 50 min, 0.1% TFA) to afford Ac-WF-NH_2 (17.68 mg, 45%). **LR-MS (+ESI)** m/z = 393.20 $[M+H]^+$.

Synthesis of Ac-WG-NH_2

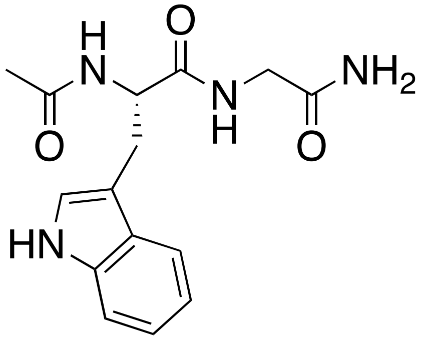

Fmoc-Gly-OH was loaded onto Rink Amide resin (100 µmol) as per the general procedures. Fmoc-Trp(Boc)-OH was then coupled for 2 h as described in the general procedures, including a final capping step for *N*-acetylation. The resin-bound peptide was treated with TFA, tri*iso*propylsilane and water (18:1:1 v/v/v, 5 mL) for cleavage from the resin and removal of protecting groups, as detailed in the general procedures. Crude peptide was purified by reversed-phase HPLC (0 to 50% B over 50 min, 0.1% TFA) to afford Ac-WG-NH_2 (8.9 mg, 29%). **LR-MS (+ESI)** m/z = 303.15 $[M+H]^+$.

Synthesis of Ac-WK-NH_2

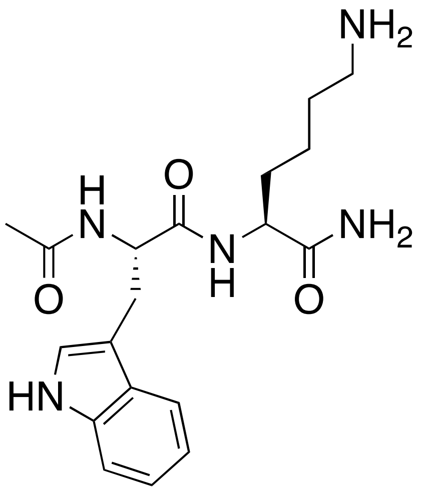

Fmoc-Lys(Boc)-OH was loaded onto Rink Amide resin (100 µmol) as per the general procedures. Fmoc-Trp(Boc)-OH was then coupled for 2 h as described in the general procedures, including a final capping step for *N*-acetylation. The resin-bound peptide was treated with TFA, tri*iso*propylsilane and water (18:1:1 v/v/v, 5 mL) for cleavage from the resin and removal of protecting groups, as detailed in the general procedures. Crude peptide was purified by reversed-phase HPLC (0 to 50% B over 50 min, 0.1% TFA) to afford Ac-WK-NH_2 (3.4 mg, 9%). **LR-MS (+ESI)** m/z = 374.1 $[M+H]^+$.

Synthesis of Ac-WS-NH_2

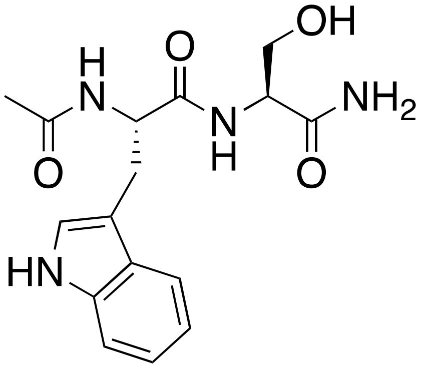

Fmoc-Ser(tBu)-OH was loaded onto Rink Amide resin (100 µmol) as per the general procedures. Fmoc-Trp(Boc)-OH was then coupled for 2 h as described in the general procedures, including a final capping step for *N*-acetylation. The resin-bound peptide was treated with TFA, tri*iso*propylsilane and water (18:1:1 v/v/v, 5 mL) for cleavage from the resin and removal of protecting groups, as detailed in the general procedures. Crude peptide was purified by reversed-phase HPLC (0 to 50% B over 50 min, 0.1% TFA) to afford Ac-WS-NH_2 (21.9 mg, 59%). **LR-MS (+ESI)** m/z = 333.15 $[M+H]^+$.

Synthesis of Ac-WV-NH_2

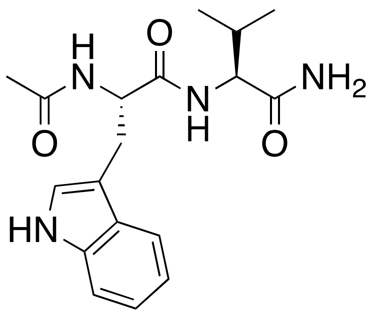

Fmoc-Val-OH was loaded onto Rink Amide resin (100 µmol) as per the general procedures. Fmoc-Trp(Boc)-OH was then coupled for 2 h as described in the general procedures, including a final capping step for *N*-acetylation. The resin-bound peptide was treated with TFA, tri*iso*propylsilane and water (18:1:1 v/v/v, 5 mL) for cleavage from the resin and removal of protecting groups, as detailed in the general procedures. Crude peptide was purified by reversed-phase HPLC (0 to 50% B over 50 min, 0.1% TFA) to afford Ac-WV-NH_2 (11.9 mg, 35%). **LR-MS (+ESI)** m/z = 345.05 $[M+H]^+$.

Synthesis of K(NFK)F

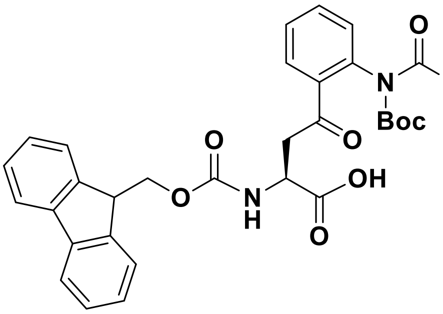

Fmoc-Trp(Boc)-OH (2.0 g, 3.8 mmol) was dissolved in 10 mL CH_2Cl_2 and cooled to -78 ℃. The resulting solution was treated with O_3 at -78 ℃ for 5 min. Me_2S (2 mL, 27.23 mmol) was then added at -78 ℃. The reaction mixture was allowed to warm to room temperature over 2 h. The reaction mixture was concentrated under vacuo and purified by flash column chromatography on silica gel (EtOAc/Hexane gradient 20-90% with 1% AcOH) to give Fmoc-NFK(Boc)-OH (2.0 g, 95 %) LRMS R_t = 3.50 min. (0 to 100 vol.% B over 5 min, 0.1 vol.% Formic Acid, λ = 214 nm) $[M+H+Na]^+$ 581.2 1H NMR (300 MHz, DMSO) δ 12.58 (s, 1H), 9.22 (d, J = 4.8 Hz, 1H), 8.01 (d, J = 7.6 Hz, 1H), 7.89 (d, J = 7.5 Hz, 2H), 7.75 – 7.56 (m, 5H), 7.41 (t, J = 7.4 Hz, 2H), 7.33 (d, J = 7.4 Hz, 3H), 4.48 (q, J = 6.2 Hz, 1H), 4.30 (d, J = 7.3 Hz, 1H), 4.23 (t, J = 4.6 Hz, 2H), 3.55 – 3.35 (m, 1H), 1.92 (d, J = 1.1 Hz, 2H), 1.37 (d, J = 7.4 Hz, 9H).

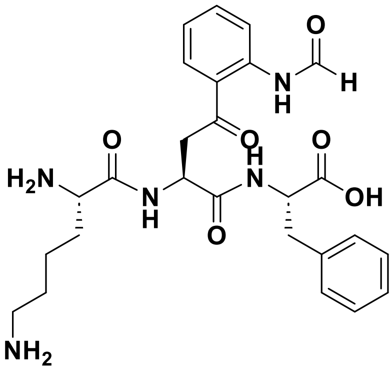

K(NFK)F was synthesised via Fmoc-strategy SPPS. The linear sequence *N'*-K(NFK)F-*C'* was generated by solid phase peptide synthesis on 2-chlorotrityl resin (102 mg, 50 µmol, capacity: 0.49 $mmolg^{-1}$) as described in the general methods. The crude linear peptide was purified by semi-preparative RP-HPLC (0 to 40 % B + 0.1 %

Formic over 40 min). The appropriate fractions were combined and lyophilised to afford **K(NFK)F** as a white solid (5.6 mg, 15%). **HPLC:** R_t = 14.89 min. (0 to 40 vol.% B over 30 min, 0.1 vol.% TFA, λ = 214 nm). 98% by UV **LRMS** (ESI+): *m/z* = 512.4 $[M + H]^+$.

Mass spectra of peptides

Ac-WG-NH2

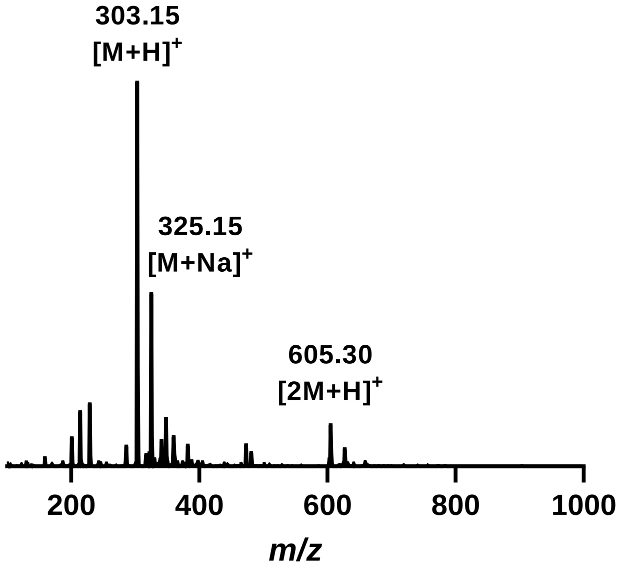

Ac-WS-NH2

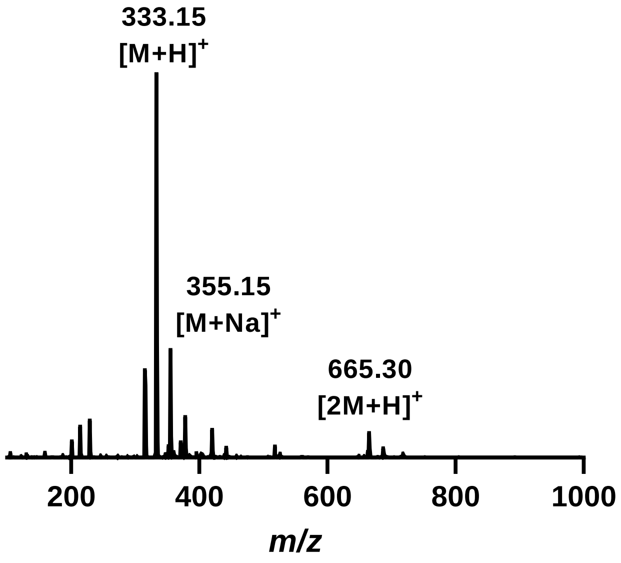

Ac-WA-NH2

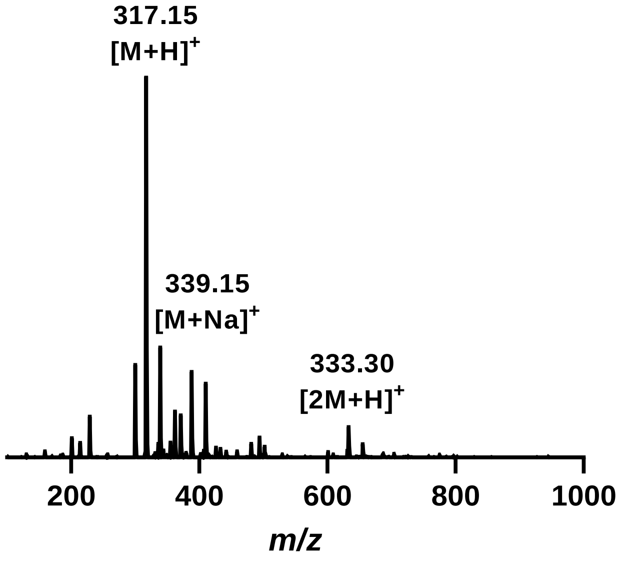

Ac-WF-NH2

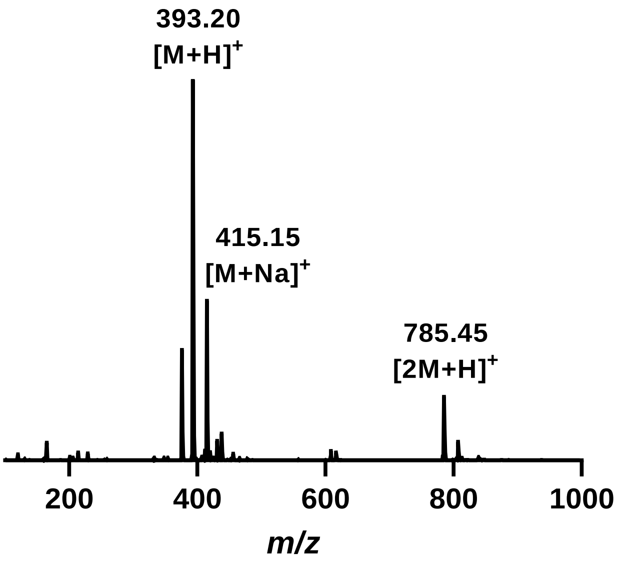

WF-NH2

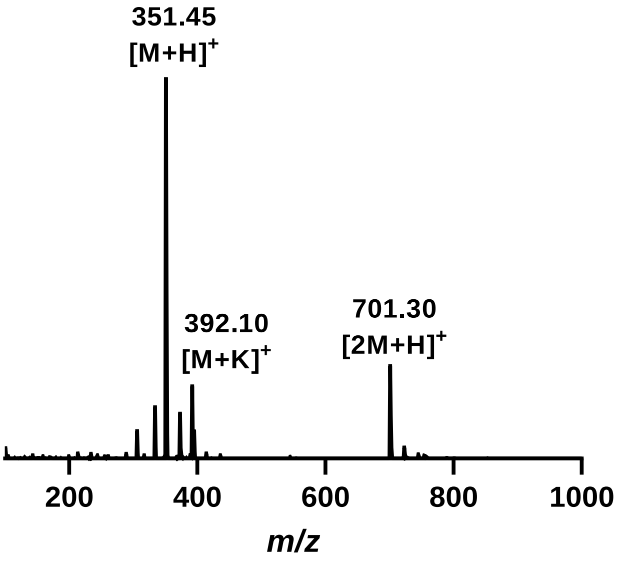

WV-NH2

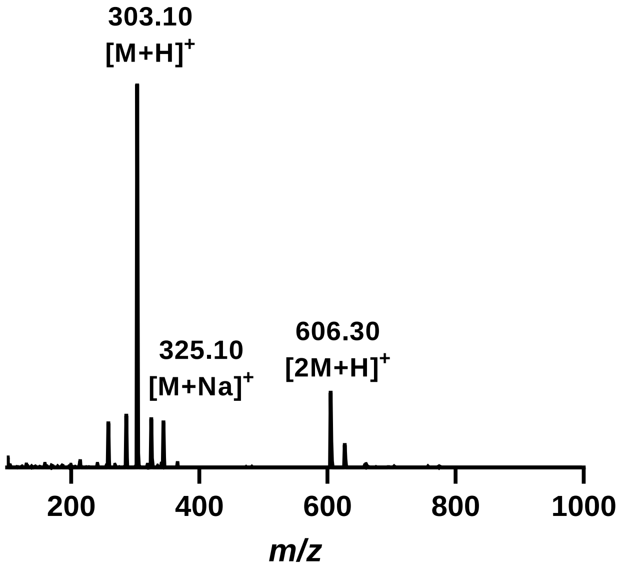

Ac-WV-NH2

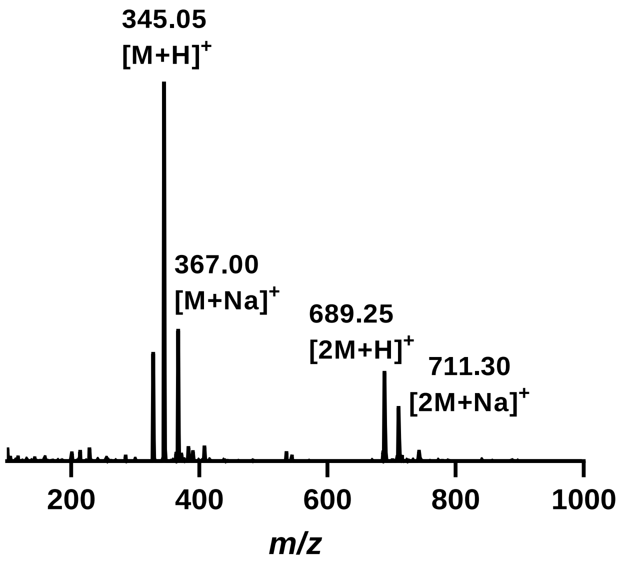

Ac-WK-NH2

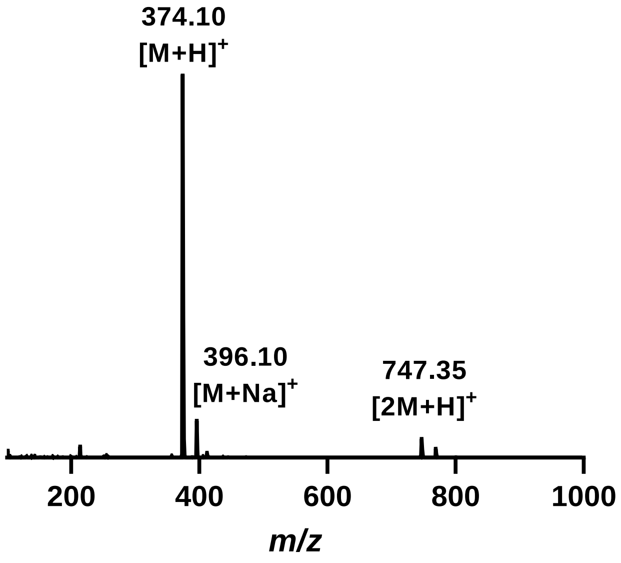

K(NFK)F

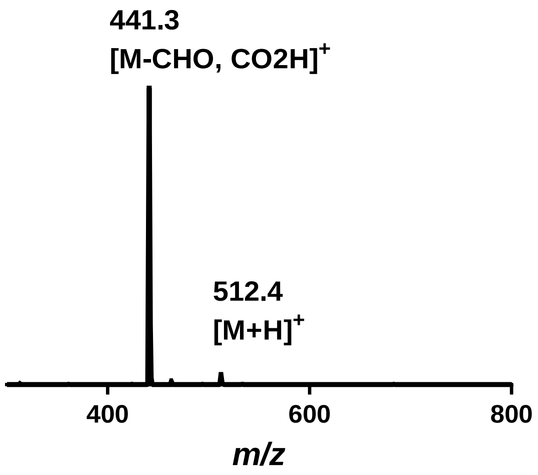

Supporting Data

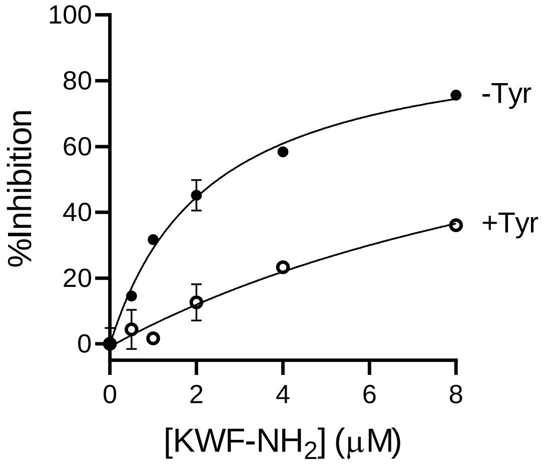

Fig. S1. KWF inhibits HOCl production by MPO. MPO (20 nM) was incubated in the presence or absence of 10 µM tyrosine (Tyr) and increasing concentrations of amidated KWF-NH_2 for 4 min at 20–22 °C in 10 mM phosphate buffer pH 7.4 containing 5 mM taurine, 140 mM chloride and 50 µM H_2O_2. The reaction was stopped by addition of 20 µg/ml catalase and the amount of taurine chloramine (TauCl) was quantified calorimetrically in a plate reader using TMB and iodide as described before[74]. A standard curve (0–50 µM TauCl) was used to express the inhibitor effect in relation to the full MPO (± tyrosine) reaction in the absence of peptide. Data points are mean ± SD of technical duplicates. A curve was fitted in GraphPad Prism (Version 8.2.1, [agonist] vs. response, three parameters). The IC_{50} for KWF-NH_2 was 2.3 µM and >>8 µM in the absence and presence of tyrosine, respectively. Loss of inhibition in the presence of tyrosine indicates that KWF-NH_2 is a competitive substrate of compound I that reduces it to compound II, which can be subsequently reduced by tyrosine to recycle the active enzyme and restore HOCl production.

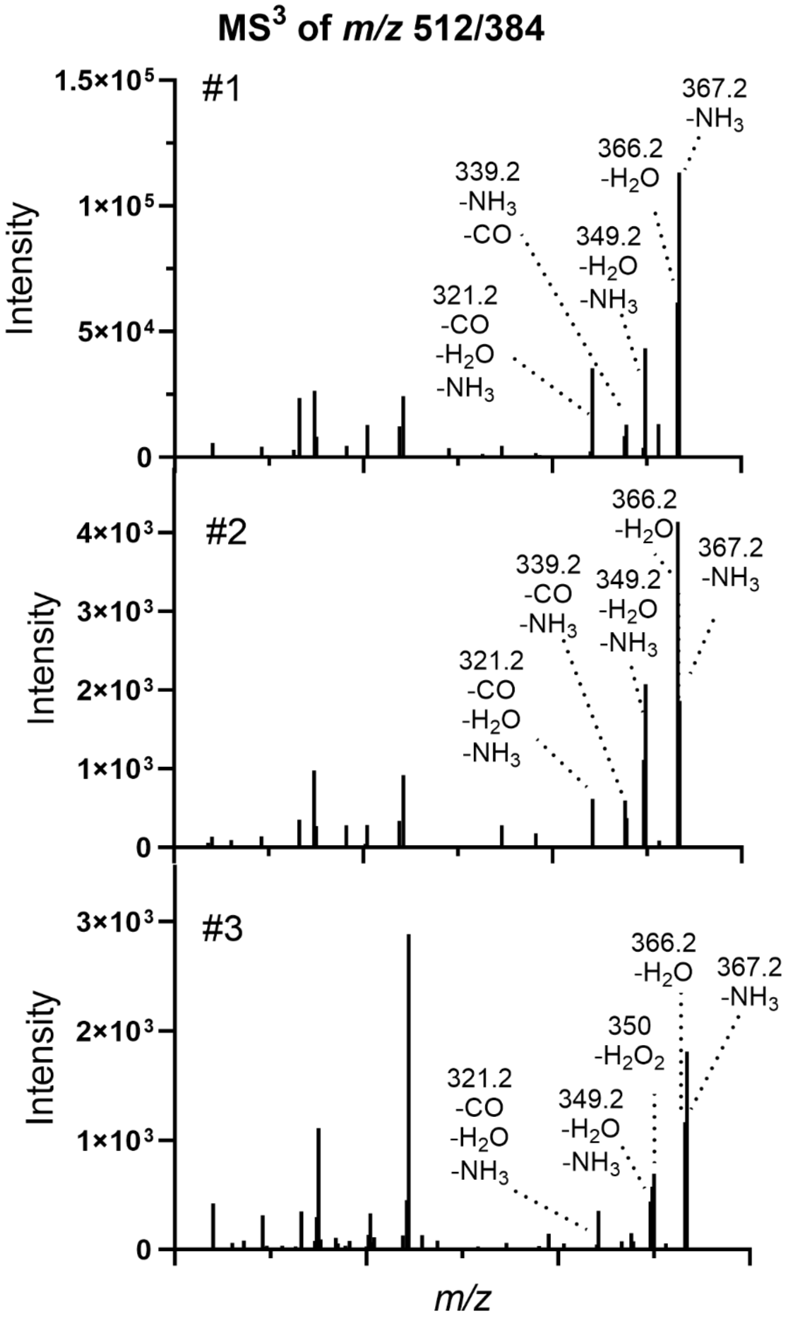

Fig. S2. MS^3 spectra for dioxygenated KWF. The y_2 (*m/z* 384.2) ions in the MS^2 spectra of all three *m/z* 512 species representing KWF+32 (shown for the major product in Fig. 2F) were subjected to further fragmentation. Representative MS^3 spectra are shown. The numbers #1, #2 and #3 represent the products shown in Fig 2D.

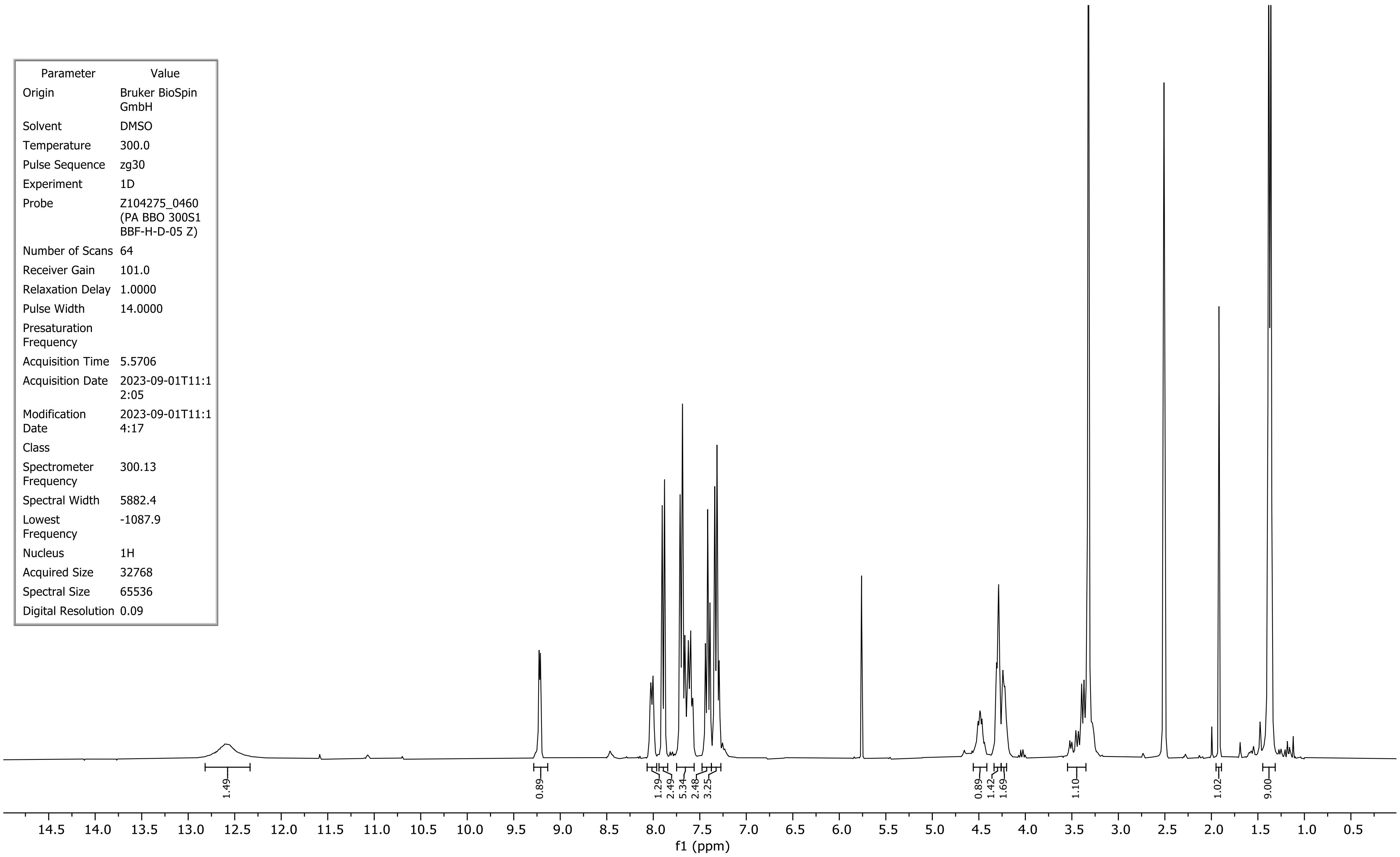

Parameter	Value
Origin	Bruker BioSpin GmbH
Solvent	DMSO
Temperature	300.0
Pulse Sequence	zg30
Experiment	1D
Probe	Z104275_0460 (PA BBO 300S1 BBF-H-D-05 Z)
Number of Scans	64
Receiver Gain	101.0
Relaxation Delay	1.0000
Pulse Width	14.0000
Presaturation Frequency	
Acquisition Time	5.5706
Acquisition Date	2023-09-01T11:12:05
Modification Date	2023-09-01T11:14:17
Class	
Spectrometer Frequency	300.13
Spectral Width	5882.4
Lowest Frequency	-1087.9
Nucleus	1H
Acquired Size	32768
Spectral Size	65536
Digital Resolution	0.09

Fig. S3. NMR spectrum of Fmoc-NFK(Boc)-OH.

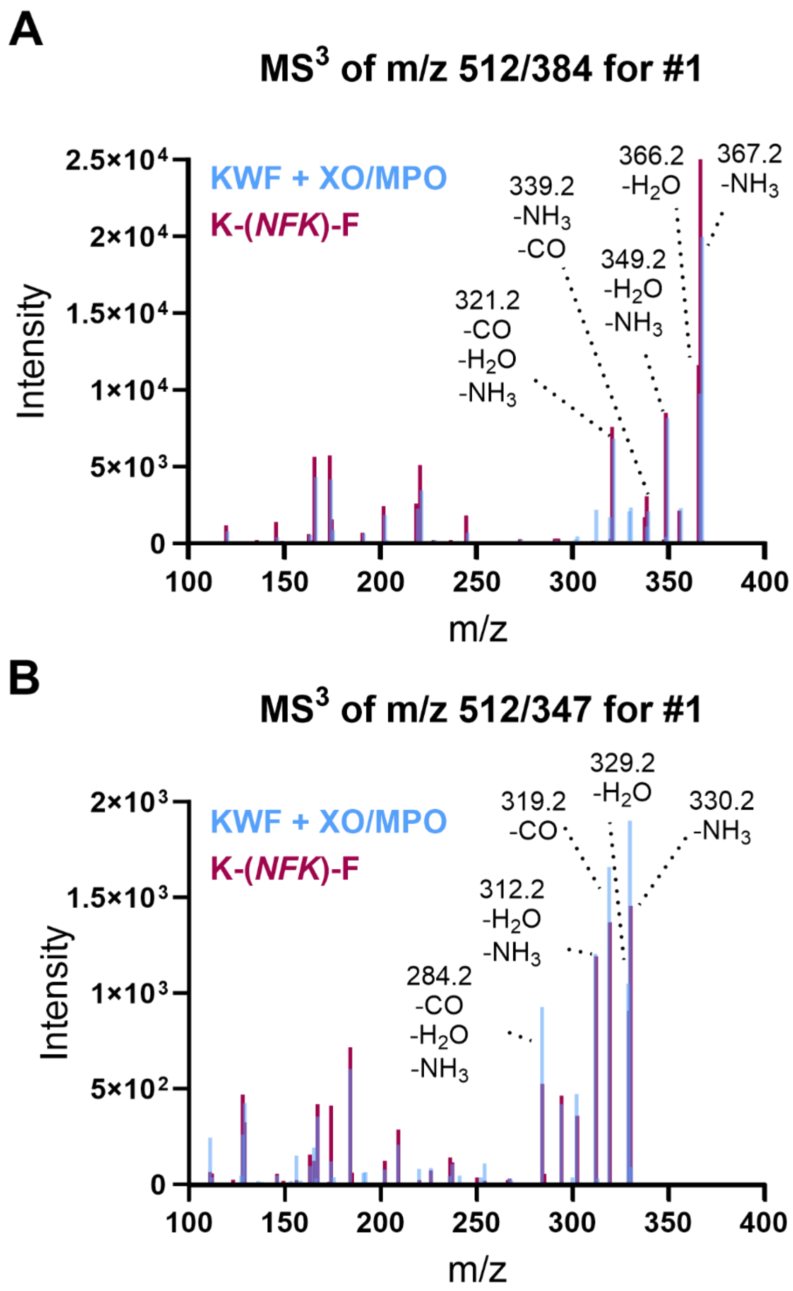

Fig S4. Overlaid MS^3 spectra for the main dioxygenation product of KWF and standard K-*NFK*-F. KWF was exposed to MPO and superoxide generated by xanthine oxidase as described in Fig. 2C, then MS^3 spectra were acquired for the **A)** y_2 and **B)** b_2 ions of the major deoxygenation product (#1 in Fig. 2D) and are shown in blue. Superimposed in red are analogous MS^3 spectra acquired for the K-(*NFK*)-F standard.

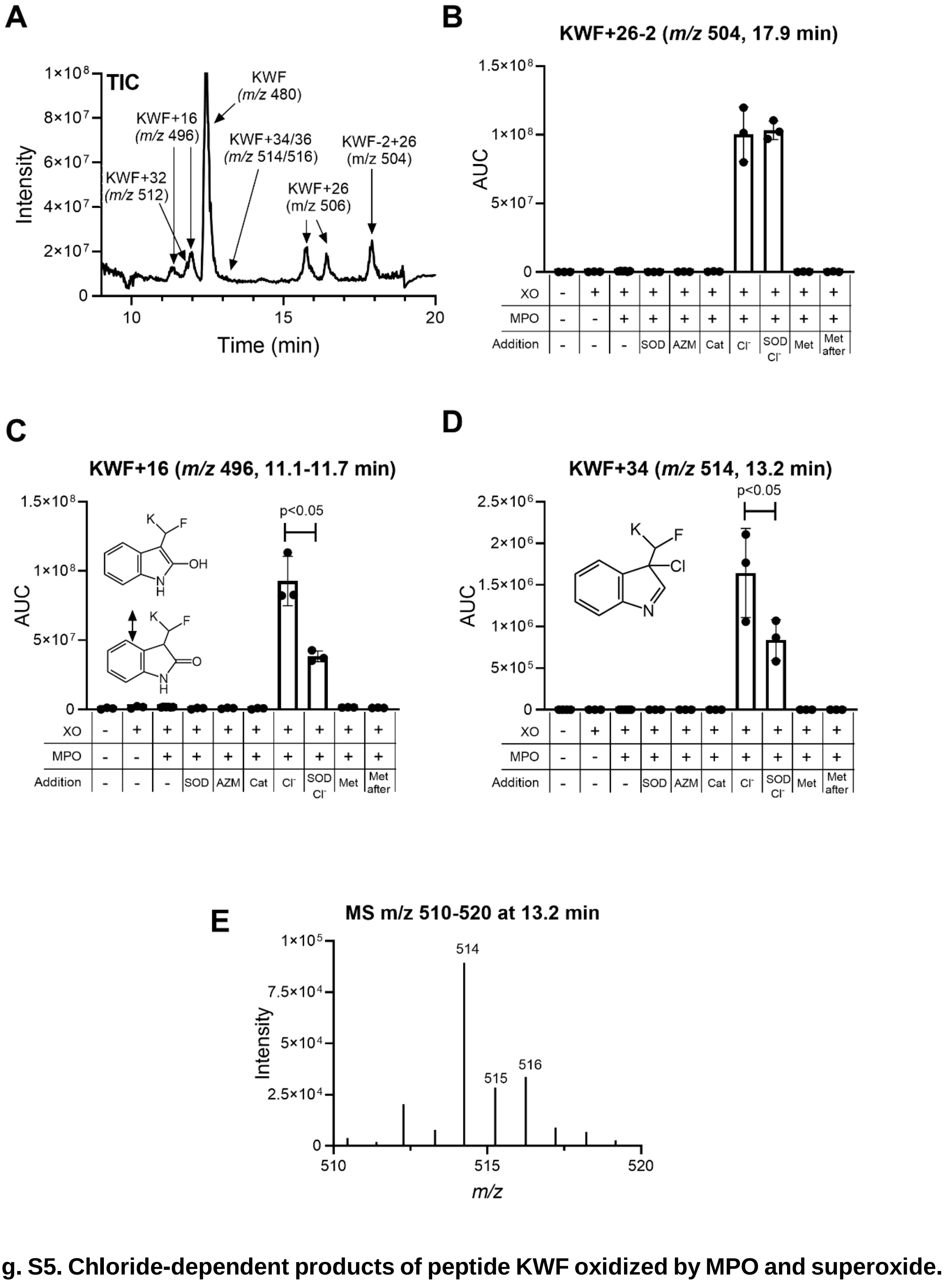

Fig. S5. Chloride-dependent products of peptide KWF oxidized by MPO and superoxide. Reaction conditions were as in Fig. 2C, but also contained the following inhibitors: superoxide dismutase (SOD, 20 µg/ml), MPO inhibitor AZM198 (10 µM), catalase (Cat, 20 µg/ml), sodium

chloride (Cl^-, 100 mM), methionine (Met, 1 mM), histidine (His, 1 mM). **A)** A representative total ion chromatogram (TIC) is shown for the system containing chloride. **B-D)** The AUC for *m/z* 504, *m/z* 496 (as a collective of peaks eluting at 11.-11.7 min) and *m/z* 514 was determined for all reaction systems. Data points represent the AUC from independent experiments and the bar the mean ± SD. A statistical difference ($p<0.05$) between the two chloride-containing systems was determined by an unpaired, two-tailed t-test. **E)** MS spectrum of KWF+34 (*m/z* 514) and KWF+36 (*m/z* 516) eluting at 13.2 min showing the characteristic 3:1 isotopic ratio of a chlorinated product.

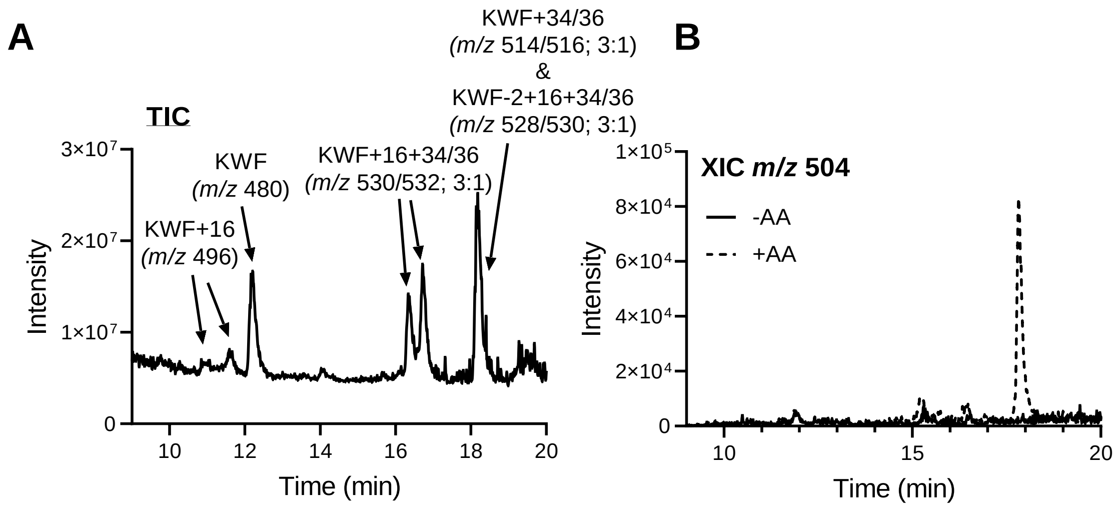

Fig. S6. Reaction products of KWF and HOCl. A) KWF (100 µM) was treated with reagent HOCl (100 µM) in 100 mM phosphate, pH 7.4 with 10 µM DTPA, incubated for 30 min at 22–24 °C, then injected for LC-MS analysis as described in Material and Methods. A representative total ion chromatogram (TIC) of three independent experiments is shown. **B)** KWF was treated with HOCl at a ratio of 1:1 in the absence (-, solid line) or presence (+, dashed line) of acetaldehyde (AA, 10 mM) and injected for LC-MS analysis. A representative extracted ion chromatogram (XIC) of two independent experiments is shown for *m/z* 504.
